# 1,3,5-Triaryl-1,3,5-Triazinane-2,4,6-Trithiones: Synthesis, Electronic Structure and Linear Optical Properties

**DOI:** 10.3390/molecules25225475

**Published:** 2020-11-23

**Authors:** Ismaël Rabouel, Nicolas Richy, Anissa Amar, Abdou Boucekkine, Thierry Roisnel, Olivier Mongin, Mark G. Humphrey, Frédéric Paul

**Affiliations:** 1 ISCR (Institut des Sciences Chimiques de Rennes), Université de Rennes, CNRS, UMR 6226, 35000 Rennes, France; ismael.rabouel@univ-rennes1.fr (I.R.); nicolas.richy@univ-rennes1.fr (N.R.); thierry.roisnel@univ-rennes1.fr (T.R.); olivier.mongin@univ-rennes1.fr (O.M.); 2 Département de Chimie, Faculté des Sciences, Université Mouloud Mammeri, 15000 Tizi-Ouzou, Algeria; amaranissa2005@yahoo.fr; 3 Faculté de Chimie, Université des Sciences et de la Technologie Houari-Boumediene, 16111 Bab-Ezzouar, Algeria; 4 Research School of Chemistry, Australian National University, Canberra, ACT 2601, Australia

**Keywords:** thioisocyanurates, isocyanurates, thionation, DFT calculations, cyclic thioureas, linear optics, cyclic urea

## Abstract

The synthesis of four new 1,3,5-triaryl-1,3,5-triazinane-2,4,6-trithione derivatives (thioisocyanurates) and two new partially thionated analogues from the corresponding 1,3,5-triaryl-1,3,5-triazinane-2,4,6-triones (isocyanurates) is reported, together with their spectroscopic properties. DFT calculations and comparison with the corresponding isocyanurates evidence the impact of the oxygen-for-sulfur replacement on the electronic structure and linear optical properties of these heterocycles. A bathochromic shift of the absorption bands and more efficient quenching of the fluorescence was observed.

## 1. Introduction

**Scheme 1:**
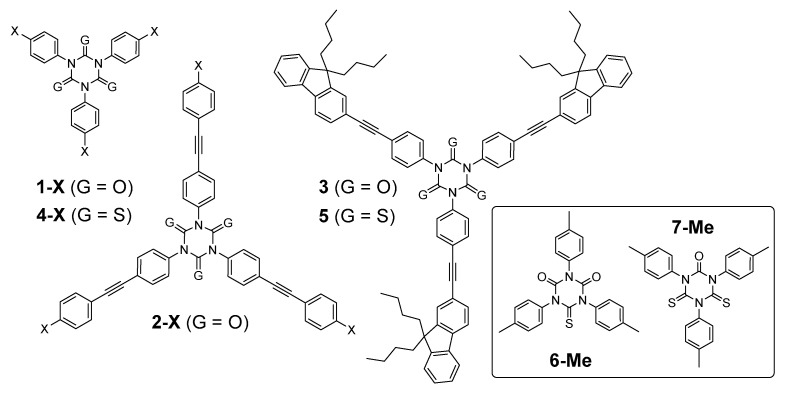
Molecular structures of known isocyanurates and of some the targeted analogues.

Triaryl-1,3,5-triazinane-2,4,6-triones (triarylisocyanurates) such as **1-X** are cyclotrimers known since the pioneering work of Hofmann in 1870 (Scheme 1) [1]. The nonlinear optical (NLO) properties of these octupolar [2] heterocycles have previously attracted our attention. π-Extended derivatives such as **2-X** were shown to possess good two-photon absorption (2PA) cross-sections and were often highly fluorescent; this is particularly the case for derivatives featuring strongly electron-rich terminal substituents X [3]. More recently, related triarylisocyanurates such as **3** were also examined for their 2PA cross-section/fluorescence products [4], in the context of using these functionalized analogues as two-photon dyes for fluorescent bio-imaging purposes [5]. As the introduction of more polarizable sulfur atoms has often been demonstrated to boost the third-order NLO properties, [6,7,8] we wondered if a similar behavior would be seen in proceeding to the 1,3,5-triazinane-2,4,6-trithione (thioisocyanurate) analogues of **1-X** and, in particular, to the model compound **5**, the thioisocyanurate analogue of **3**.

We noted that a versatile synthetic access to the extended thioisocyanurates such as **5** was lacking and, more generally, that an extensive characterization of the shorter homologue 1,3,5-triaryl-1,3,5-triazinane-2,4,6-trithiones **4-X** was needed. Indeed, whereas triphenyl-1,3,5-triazinane-2,4,6-trione (**4-H**) has been reported several times in the literature [9,10,11,12,13,14], and indeed characterized by X-ray diffraction [13], to the best of our knowledge, no further characterization data have been reported for this compound apart from the melting point, while data for other *para*-functionalized analogues **4-X** are totally absent. Furthermore, although **4-H** was, in principle, accessible in a straightforward fashion from phenyl thioisocyanate by cyclotrimerization using various catalysts [9,11,12,13], none of the reactions [11,13] provided access to this compound in our hands, so we eventually resorted to another synthetic approach based on isocyanurate thionation [15].

We therefore report herein a synthetic approach to **4-H** and the new related derivatives **4-X** (X = Me, Br, I), along with the extensive characterization of all these molecules. **4-I** has been used to synthesize the extended 1,3,5-triazinane-2,4,6-trithione derivative **5**, for which full characterization data are reported. The properties of the partially thionated compounds **6-Me** and **7-Me** have also been investigated. Finally, we discuss the impact of the oxygen-for-sulfur exchange on the electronic structure and linear optical properties of these heterocycles.

## 2. Results and Discussion

### 2.1. Synthesis

**Scheme 2:**
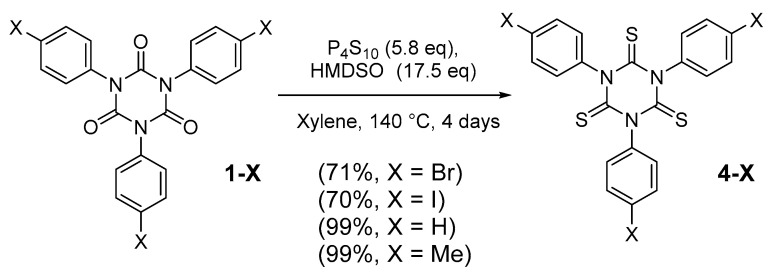
Synthesis of **4-X** derivatives.

The targeted thioisocyanurates **4-X** (X = Br, I, H, Me) were isolated in one step from their corresponding isocyanurate analogues in good yields (70–99%) by using an improved experimental protocol (Scheme 2) derived from that previously used by Mazzanti et al. to access the mono-thionated derivatives of tri(2-tolyl)isocyanurate [15].

**Figure 1:**
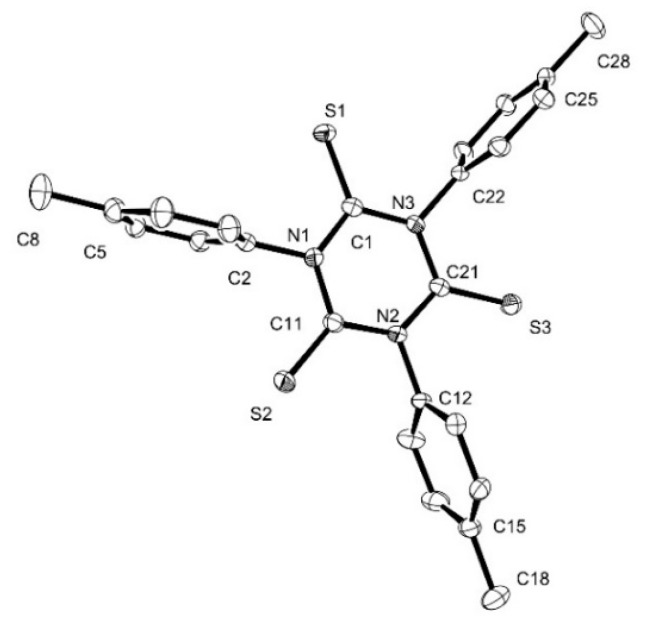
ORTEP representation of **4-Me** at the 50% probability level. Hydrogen atoms have been omitted for clarity. Selected distances [Å]: S1–C1 1.650(2), S2–C11 1.637(2), S3–C21 1.637(2), C1–N1 1.384(2), C11–N1 1.391(3), C11–N2 1.388(3), C21–N2 1.390(2), C1–N3 1.385(2), C21–N3 1.396 (2), N1–C2 1.456(2), N2–C12 1.461(2), N3–C22 1.458(2).

Compared to its success with the more sterically congested isocyanurates [15], this protocol did not work as efficiently for **1-Me**, instead affording significant quantities of unreacted starting material. Changing the CH_2_Cl_2_ solvent for xylenes and using harsher conditions for four days gave the desired cyclotrimer **4-Me** in modest yield (6%), along with the partly thionated derivatives **6-Me** (20%) and **7-Me** (24%). A larger excess of P_4_S_10_ and hexamethyldisiloxane (HMDSO) in refluxing xylene effected the complete reaction, providing a selective access to the desired tri-thionated product in four days without the need for chromatographic purification. Through these means, **4-Me** and related analogues **4-X** (X = H, Br, I) could be isolated in good yields from the corresponding isocyanurates **1-X**. Most of these compounds were characterized by the usual means (^1^H- and ^13^C-NMR, FTIR), and by crystallography in the case of **4-Me** (Figure 1) [16]. The diagnostic ^13^C-NMR resonance of the carbonyl groups of the isocyanurate in **1-Me** (ca. 148 ppm) was upfield-shifted (to ca. 178 ppm) upon replacement of the oxygen by sulfur (Table 1). As expected, this particular carbon nucleus has a chemical shift in the range reported for the C=S nucleus in thioureas [17,18,19,20].

**Scheme 3:**
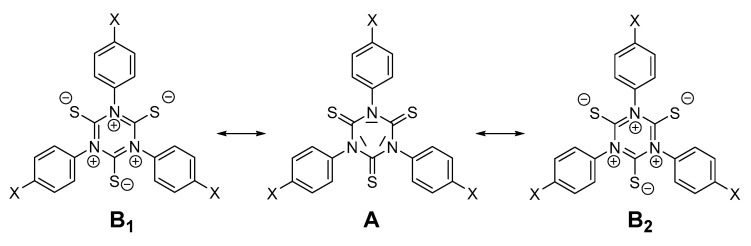
Mesomerism in **4-X** compounds.

The crystallographic structure of **4-Me** (Figure 1, Table S1 in Supplementary Materials) clearly revealed the presence of the three sulfur atoms and of a nearly planar central heterocyclic core (torsion angles of less than 12° between the C=S bonds) [21]. The mean values for the C–S bond lengths (1.644 ± 0.007 Å) were in the range expected for double bonds [22]. These data are in accordance with the structural data previously reported for **4-H** (C–S, 1.65 ± 0.04 Å) [13], the only thioisocyanurate structurally characterized so far. The C=S bond lengths of **4-Me** were slightly longer than those of aromatic thioketones (1.61 Å) [23], but shorter than those of typical thioureas (1.68 ± 0.02 Å), whereas the (S)C–N bond lengths were significantly shorter (ca. 1.39 Å) than typical single N–C_Ar_ bonds (1.48 Å) [22,24]. Along with the overall planarity of the thioisocyanurate ring, these bonding features indicate the existence of some mesomerism between the dominant neutral valence-bond (VB) form (**A**) and the two other VB structures (**B_1_** and **B_2_**) (Scheme 3), consistent with previous observations for isocyanurates [3].

**Figure 2:**
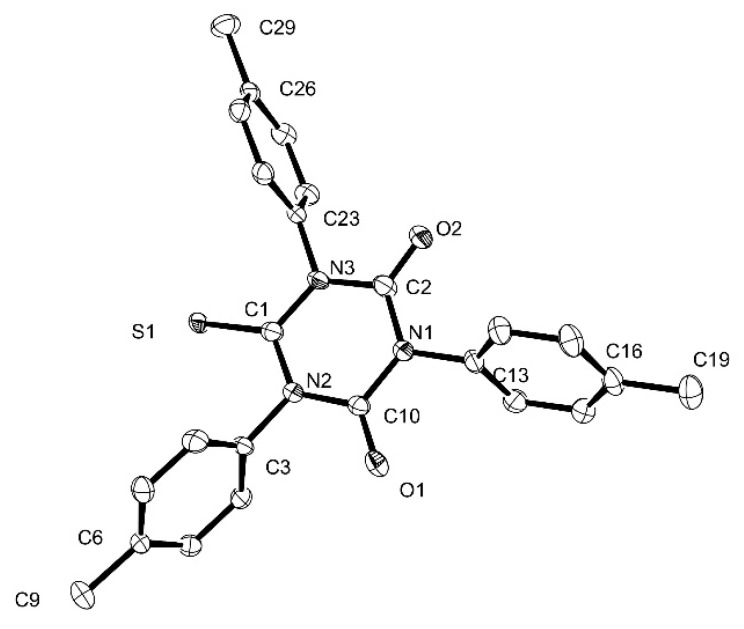
ORTEP representation of **6-Me** at the 50% probability level. Hydrogen atoms have been omitted for clarity. Selected distances [Å]: O1–C10 1.242(4), O2–C2 1.203(6), S1–C1 1.666(4), C10–N1 1.389(4), C2–N1 1.388 (5), C1–N2 1.386(4), C10–N2 1.393(4), C1–N3 1.385(4), C2–N3 1.339(5), N1–C13 1.448(4), N2–C3 1.456(4), N3–C23 1.456(4).

As mentioned earlier, when the reaction with **1-Me** was run at a lower temperature, mixtures of mono-, bis-, and tris-thionated derivatives were formed, providing us with an opportunity to isolate the new derivatives **6-Me** and **7-Me** after chromatographic separation. Apart for one example [15], these are the first “S,O-mixed” thioisocyanurates ever isolated. Although their yields were poor (20% and 24%, respectively), these compounds were fully characterized by the usual means as well as by crystallography in the case of **6-Me** (Figure 2, Table S1) [25]. Despite some positional disorder, the presence of one C–S bond was clearly apparent in the molecular structure of this compound (1.66 ± 0.01 Å). The two C–O bonds (1.22 ± 0.02 Å) were significantly shorter, and were in the range of those reported for the carbonyl bonds of **1-Me** (1.21 ± 0.01 Å) [26,27]. The central heterocycle was nearly coplanar.

**Scheme 4:**
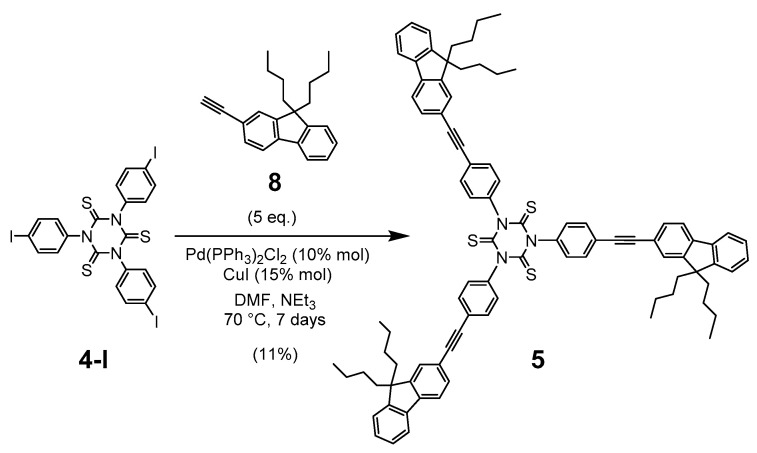
Synthetic routes toward the thioisocyanurate **5** featuring an extended π-manifold.

To access the extended thioisocyanurate derivative **5**, a Sonogashira coupling reaction [28] between the previously isolated tris(4-iodophenyl)thioisocyanurate precursor **4-I** and the known alkyne **8** [29] was performed (Scheme 4), analogous to that with related isocyanurates [4]. After tedious chromatographic separation, the desired product **5** was eventually isolated in poor yield (11%) and was characterized (Table 1).

### 2.2. Vibrational Properties

As the ν(C=O) modes provide a simple means to characterize isocyanurates [3], we looked for the vibrational signature of the ν(C=S) modes in thioisocyanurates. The C=S vibrator has a weaker bond strength and is less polar than the C=O vibrator, so its stretching mode is expected to be less energetic and less intense in the infrared (IR) than the corresponding ν(C=O) mode. Accordingly, values in the 650–950 cm^−1^ range have been proposed for ν(C=S) in tertiary thioamides [31] or thioureas, [32,33,34] (i.e., below the range traditionally proposed for this mode in thioketones or secondary thioamides (1200–1000 cm^−1^)) [35,36,37]. In thioamides, thiocarbamates, and thioureas, it has also been observed that this mode has a strong tendency to couple with the ν(C–N) mode(s) of the nearby nitrogen atom(s), and so it does not quite correspond to a “pure” C=S stretching motion [35,36]. As a result, four characteristic bands (I–IV) are usually reported for secondary thioureas, the ν(C=S) motion contributing to the three at lower energy (II–IV). This contribution has increasing weight upon decrease in energy of the band, so the lowest energy one (IV) is often reported as the ν(C=S) stretch. Although “pure” ν(C=S) modes should be Raman-active due to the strong polarizability of sulfur, their unambiguous identification is often problematic due to this vibrational coupling [33].

**Figure 3:**
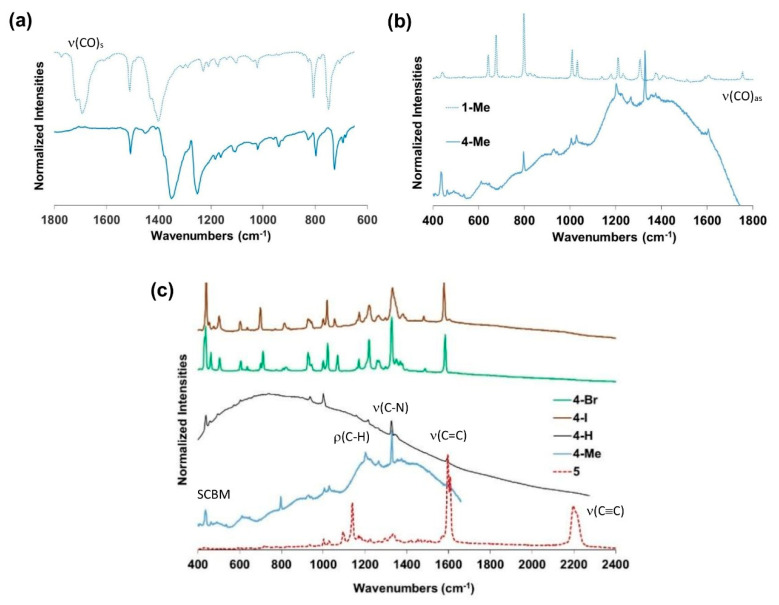
(**a**) IR (ATR) and (**b**) Raman spectra of **1-Me** vs. **4-Me** in the solid state (20 °C). The symmetric *A* and asymmetric *E* ν(CO) modes are indicated on the spectra of **1-Me**. (**c**) Raman spectra of **4-X** (X = Br, I, H, Me) and **5** in the solid state (20 °C). Selected bands for **4-Me** are attributed to specific vibrational modes based on DFT calculations. The symmetric “breathing mode” of the core coupled with the C=S stretch is indicated as “SCBM”.

The effect of oxygen-for-sulfur replacement at the isocyanurate core on these vibrations was initially studied using the derivatives **1-Me**, **6-Me**, **7-Me**, and **4-Me**, and then extended to the other thioisocyanurate derivatives **4-X** (X = Br, I, H) and **5** (Figures S9–S11). Along the first series, the specific vibrational signature of the carbonyl groups (ca. 1710 cm^−1^ in the IR (*E*-type mode) and 1770 cm^−1^ (*A*-type mode) in the Raman) [3] disappeared in a stepwise fashion upon progressing from **1-Me** to **4-Me**. Concomitantly, new absorption bands appeared in the 1650–400 cm^−1^ spectral range. Based on DFT computations (see DFT section) [32], these changes cannot be simply attributed to the appearance of new ν(C=S) modes, but instead originate from more complex behavior involving the shift and intensity-enhancement of other modes in the thionated derivatives. Thus, for all thioisocyanurates, two diagnostic IR-active modes around 1330 cm^−1^ and 1250 cm^−1^ can be identified, which most likely correspond to bands II and III of the secondary thioureas (Table 1). In contrast, the two ν(C=S) symmetric (*A*-type) and antisymmetric (*E*-type) mode(s) cannot be easily discriminated from other vibrational modes in the IR spectra and are thus not diagnostic. However, in thioisocyanurates, we can see (with the help of the DFT calculations) that the symmetric breathing mode of the central heterocycle becomes strongly admixed with the symmetric C=S stretch, a feature rendering this low-energy mode Raman-active and characteristic of the thionated core. This intense Stokes band was located at 436 cm^−1^ in the Raman spectrum of **4-Me** (Figure 3) and, by analogy, at ca. 450 cm^−1^ in the spectra of the other thioisocyanurates **4-X** or **5** (Table 1). Finally, according to the DFT calculations, the ν(C=S) modes of **6-Me** and **7-Me** were not easily distinguishable from other vibrational modes in the same spectral range and can therefore not be considered as reliable spectroscopic markers for these compounds.

### 2.3. Linear Photophysical Properties

The electronic absorption and emission spectroscopic properties of the various thioisocyanurates **4-X** and **5** were next determined (Table 2) and compared to those of their known isocyanurate analogues **1-X** and **3**.

**Figure 4:**
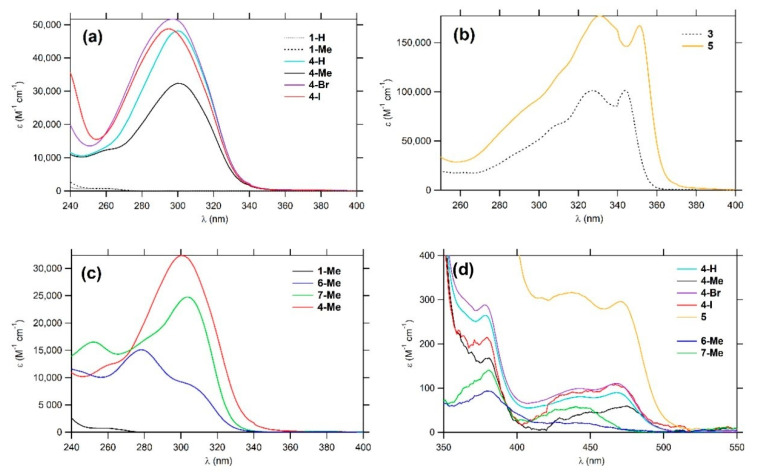
UV-vis spectra for **1-H**, **1-Me**, and **4-X** (X = Br, I, H, Me) (**a**), **3** and **5** (**b**), **1-Me**, **6-Me**, **7-Me** and **4-Me** (**c**), and the details of the 350–600 nm spectral region for **4-X** (X = Br, I, H, Me) and **5** (**d**) in CH_2_Cl_2_ (20 °C).

The spectra of the shorter derivatives **4-X** possessed a strong absorption in the UV region at ca. 300 nm (Figure 4a–c), which corresponds to a strongly allowed π*← π transition having a weak charge-transfer (CT) character from the peripheral arms toward the central core (see DFT computations) [3]. This transition is not solvatochromic for **4-X** and **5** (Figure S12 and Table S2). Thus, when comparing **1-Me**, **6-Me**, **7-Me**, and **4-Me**, the stepwise replacement of oxygen atoms by sulfur led to a progressive bathochromic shift of this transition (Figure 4c), resulting in a net shift of ca. 60 nm (ca. 8300 cm^−1^) for this absorption between **1-X** and **4-X** (X = Br, I, H, Me). The yellow color of these compounds in solution was due to the presence of a small band at ca. 470 nm corresponding to a partially forbidden transition within the central core (Figure 4d). Similar transitions have previously been identified for isocyanurates, but at higher energies (around 280–320 nm), and as shoulders on the low-energy side of the first absorption band for **1-X** derivatives [3]. Upon extension of the conjugated arms, both transitions shifted bathochromically and appeared at 351 nm and 470 nm in **5** (Figure 4b,d).

None of these new thioisocyanurates were emissive in solution at 25 °C, but at 77 K in ethanol glasses (Table S3), a weak luminescence around 515 nm (*Φ*_F_ not measurable) was observed for **4-Me** and **5**, apparently originating from the shoulder at ca. 460–470 nm, while structured phosphorescence (*Φ*_P_ also not measurable [38]) was detected at ca. 393 nm with **1-Me** (Figure S13).

### 2.4. Density Functional Theory (DFT) Calculations

Density functional theory (DFT) calculations were performed on compounds **1-Me**, **4-Me**, **6-Me**, **7-Me**, and **5′**. The last mentioned was similar to **5**, but the *n*-butyl chains of **5** were replaced by methyl groups to simplify the calculations. For all of these compounds, the three peripheral arms adopted a nearly perpendicular conformation relative to the central phenyl plane after geometry optimization in CH_2_Cl_2_. Apart from this, the structural parameters were close to those observed in the corresponding x-ray structures (Table S4). Atomic charges (Mulliken or NBO) and bond orders (Wiberg) support the existence of a slight mesomery involving the central core (Figure S15), as previously proposed (Scheme 3). Unsurprisingly, dipole moments with sizable “in plane” components were found for **6-Me** (1.62 D) and **7-Me** (1.40 D). These results were from the presence of sulfur and oxygen atoms at the periphery of the central heterocyclic core and, as expected, they aligned with the C=X bond (X = O, S), which is distinct from the two others (Figures S15 and S16 and Table S5). For the extended derivative **5′**, as for its isocyanurate analogue **3′** [4], essentially two types of nearly isoenergetic conformations coexist for the GS at ambient temperatures; a statistically favored one (C1) with only two arms oriented “on the same side” of the central heterocycle and a low dipole moment (Table 3) and a second one with three arms “on the same side” (C2) and with a larger dipole moment perpendicular to the central ring (2.31 D).

**Figure 5:**
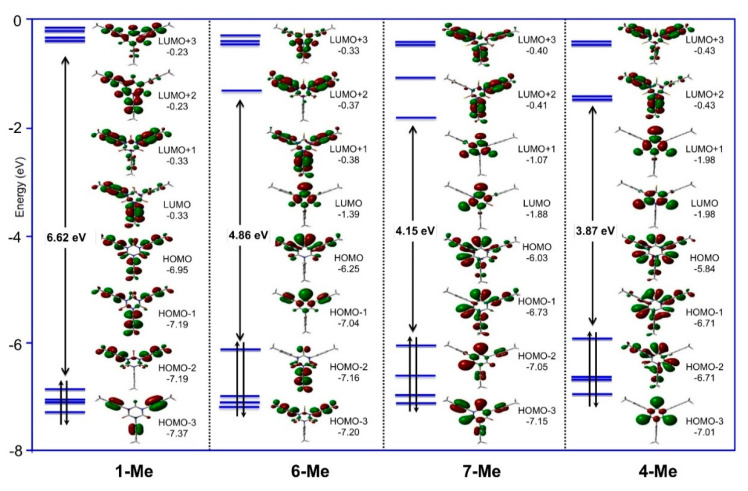
Frontier molecular orbitals involved in the lowest-energy (intense) allowed transitions for **1-Me**, **6-Me**, **7-Me**, and **4-Me** at the MPW1PW91/6-31G* level in CH_2_Cl_2_ (isocontour 0.03 [e/bohr^3^]^1/2^).

**Figure 6:**
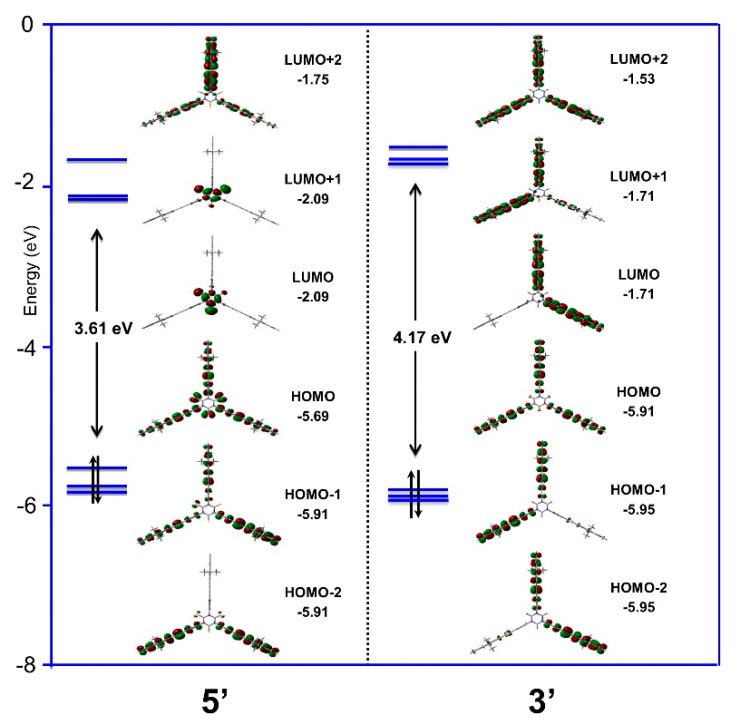
Frontier molecular orbitals involved in the lowest-energy (intense) allowed transitions for **3′** and **5′** in conformation C1 at the MPW1PW91/6-31G* level in CH_2_Cl_2_ (isocontour 0.03 [e/bohr^3^]^1/2^).

The stepwise replacement of oxygen by sulfur along the series **1-Me**, **6-Me**, **7-Me**, and **4-Me** allowed us to probe the impact of this compositional change on the electronic structure of these compounds (Figure 5). In each sulfur-containing derivative, the frontier molecular orbitals (FMOs) from HOMO-1 to LUMO+1 were much more strongly located on the central core than in **1-Me**. Furthermore, the LUMO (**6-Me**) and LUMO+1 (**4-Me** and **7-Me**) possessed a C=X π*-antibonding character, while the HOMO and HOMO-1 had a C=X π-bonding character (X = O, S). Compared to their isocyanurate analogue **1-Me**, these new FMOs were respectively lower and higher in energy. Thus, they decreased the HOMO-LUMO gap of the molecule, which consequently followed the order **1-Me** > **6-Me** > **7-Me** and **4-Me** (i.e., correlated with the increasing number of sulfur atoms in the molecule). A similar comment can be made for the LUMO and LUMO+1 of the extended thioisocyanurate derivative **5’** (Figure 6), which also had a smaller HOMO-LUMO gap than its isocyanurate analogue **3**. Comparison with **4-Me** revealed that part of this decrease in **5′** originated from the increase in the π-manifold on the peripheral arms. As a result, electronic transitions involving these particular FMOs possessed a marked directional CT character “toward” or “from” the central ring. Simulation of the vibrational properties of **1-Me**, **4-Me**, **6-Me**, and **7-Me** revealed that the ν(C=S) modes strongly coupled with other vibrational modes of similar symmetry, namely ν(C–N) or ρ(C_Ar_–H). Computationally, “ν(C=S)” modes were thus identified as the vibrational modes possessing the largest weight of C=S stretching on their potential energy distribution (PED). These modes were significantly less IR- or Raman-active than the ν(C=O) modes and arose at lower energies (1297 cm^−1^ for **6-Me**, 1295 and 1261 cm^−1^ for **7-Me**, 1271 cm^−1^ (asymmetric doubly degenerate *E*-mode) and 1230 (symmetric *A*-mode) for **4-Me** (Table S6)). In the absence of calculations, all of these low intensity modes would be difficult to distinguish from other vibrational modes in the spectra of real molecules. Fortunately, the ν(C=S) symmetric mode of **4-Me** coupled with the “breathing mode” of the central heterocycle and gave rise to an intense Raman-active mode at 453 cm^−1^ in a spectral region where few Stokes lines were present, thereby providing a diagnostic ν(C=S)-related mode for thioisocyanurates.

The TD-DFT calculations reproduced the energy ordering of the experimentally observed transitions (Table S7), but their energies were slightly overestimated. While the HOMO-LUMO gaps in **1-Me**, **4-Me**, **6-Me, 7-Me**, and **5′** (Table 3) mirror the energetic trend of the experimentally observed first intense absorptions (Table 2), the calculations revealed that the first allowed band was not a HOMO-LUMO transition. This strongly allowed transition actually involves deeper-lying occupied MOs, which give rise to a symmetrical charge transfer between the periphery and the center, with a dominant π*←π character. For **4-Me** or **5′**, although the optimized GS structures did not afford rigorous *C*_3v_ symmetry, the energetic degeneracy of the two lowest energy transitions and their spatial distributions were nevertheless reminiscent of those expected for an *E←A* transition in strict *C*_3v_ symmetry. This was not the case with **6-Me** and **7-Me**, which possess lower symmetry, but again, the first intense transition(s) took place between MOs of very similar nature.

For these derivatives, there was also a set of transitions at lower energies which includes the HOMO-LUMO excitation and that have zero or very weak oscillator strengths in the optimized GS conformation, because they take place between MOs that overlap poorly [40]. These largely symmetry-forbidden transitions are the origin of the weak shoulder observed on the low-energy side of the first absorption band discussed above (Figure 4d). Indeed, as verified with **4-Me**, rotation of the peripheral *para*-tolyl groups around the N–C_A_ bonds in the GS increased the oscillator strengths of these forbidden transitions and red-shifted their energies (Figure S17); while the twisted conformers correspond to less stable conformations because of increased steric strain, they are likely to be populated in solution at 25 °C by thermal agitation [15]. Given that all of the thioisocyanurates synthesized so far (including the partially thionated derivatives **6-Me** and **7-Me**) are not fluorescent, we believe that these nearly forbidden excited states act as efficient quenchers for luminescence, promoting non-radiative deactivation via internal conversion. Oscillator strengths for emission were computed from the first allowed excited state (after vibrational relaxation) for the strongly fluorescent extended isocyanurate **3** containing 7-fluorenyl groups at the periphery as well as its non-emissive thioisocyanurate analogue **5**. Reassuringly, the calculations indicate a relative difference between these two complexes of ca. four orders of magnitude for the radiative process. This result nicely corroborates the lack of fluorescence experimentally observed for **5**, which can now be safely attributed to efficient relaxation in a non-emissive singlet state. Similar results were obtained for the other compounds (Table S8). As an additional deactivation mechanism, rapid conversion of the initially excited singlet state into a lower-lying triplet state promoted by the presence of sulfur (heavy atom effect) might also contribute to reducing the fluorescence of these compounds. The lowest triplet states were found ca. 0.2–0.4 eV below the first singlet states for the short thionated derivatives (Table S8). However, because no phosphorescence was seen for **4-Me** or **5** at low temperatures (Figure S13), the contribution played by such an additional pathway in the non-radiative deactivation process cannot be ascertained as yet.

## 3. Materials and Methods

### 3.1. General

All manipulations were carried out under an inert atmosphere of argon with dried and freshly distilled solvents (MeOH, distilled from Mg; THF, Et_2_O, and *n*-pentane, distilled from Na/benzophenone; CH_2_Cl_2_, distilled from CaH_2_). The solvents used for spectroscopic measurements were of commercial origin: MeCN, MeOH, CH_2_Cl_2_, *n*-BuOH, *n*-hexane, and 1,4-dioxane from Merck (Darmstadt, Germany, spectral grade), toluene from POCH (Gliwice, Poland, for analysis), *n*-PrOH (Aldrich, St. Louis, MO, USA, spectrophotometric grade), and glycerol (99.5%). Transmittance-FTIR spectra were recorded using a Perkin Elmer Spectrum 100 spectrometer (Waltham, MA, USA) equipped with a universal ATR sampling accessory (400–4000 cm^−1^). Raman spectra of the solid samples were obtained by diffuse scattering on the same apparatus and recorded in the 400–2500 cm^−1^ range (Stokes emission) with a laser excitation source at 633 nm. High field NMR spectra were obtained on a multinuclear Bruker 300 MHz instrument (Karlsruhe, Germany, Avance 300). Chemical shifts are given in parts per million relative to tetramethylsilane for ^1^H- and ^13^C-NMR spectra. MS analyses were performed at the “Centre Regional de Mesures Physiques de l’Ouest” (CRMPO, Université de Rennes) on high resolution Bruker Maxis 4G or Thermo Fisher Q-Extractive Spectrometers. Elemental analyses were also performed at CRMPO. Commercial reagents were used as received. Triarylisocyanurates **1-X** (X = Br, H, Me) and 9,9-dibutyl-2-ethynyl-9*H*-fluorene (**7**) were synthesized as previously described [3,29]. The synthesis of **1-I**will be reported shortly [39].

### 3.2. Synthesis of Thioisocyanurates from Isocyanurate Precursors; General Procedure

The desired 1,3,5-triaryl-1,3,5-triazinane-2,4,6-trione (0.28 mmol), P_4_S_10_ (1.62 mmol; 5.8 eq.), hexamethyldisiloxane (4.9 mmol; 1.05 mL; 17.5 eq.), and xylene (2 mL; mixture of isomers) were added to a Schlenk flask. The resultant mixture was heated at reflux for four days (ca. 140 °C). At this stage, the desired product should be present in solution (the progress of the reaction can be monitored by TLC). After cooling to room temperature, the solvent was removed under vacuum and replaced with CH_2_Cl_2_ (10 mL), water (10 mL), and NEt_3_ (200 μL). The mixture was stirred for 1 h. The two phases were separated and the aqueous layer extracted with CH_2_Cl_2_ (3 × 10 mL). The solvent was then removed from the combined organic extracts, and the solid was purified by elution through silica (CH_2_Cl_2_) to yield the desired product.

*1,3,5-Tris(4-bromophenyl)-1,3,5-triazinane-2,4,6-trithione* (**4-Br**). Following the general procedure with 1,3,5-tris(4-bromophenyl)-1,3,5-triazinane-2,4,6-trione (**1-Br**; 163 mg), the title compound was isolated as a yellow solid (126 mg). Yield: 71%. MP: 295–320 °C (Dec). HRMS: (ASAP): *m*/*z* = 639.7817 [M + H]^+^ (calc. for C_21_H_13_N_3_Br_3_S_3_: 639.7816. ^1^H-NMR (300 MHz, CD_2_Cl_2_) δ = 7.68 (d, *J* = 8.7 Hz, 6H), 7.23 (d, *J* = 8.7 Hz, 6H). ^13^C{^1^H}-NMR (75 MHz, CD_2_Cl_2_) δ = 172.1 (*C*=S), 142.5 (N*C*_Ar_), 133.1 (*C*H_Ar_), 130.1 (*C*H_Ar_), 122.9 (Br*C_Ar_*). IR (ATR, cm^−1^): ῡ = 1344 (C–N, vs), 1255 (C–N, s). Raman (neat, cm^−1^): ῡ = 1584 (C=C, s), 1327 (C–N, vs), 1220 (C–H), 438 (C=S, vs).

*1,3,5-Tris(4-iodophenyl)-1,3,5-triazinane-2,4,6-trithione* (**4-I**). Following the general procedure with 1,3,5-tris(4-iodophenyl)-1,3,5-triazinane-2,4,6-trione (**1-I**, 200 mg), the title compound was isolated as a yellow solid (150 mg). Yield: 70%. MP: 250–270 °C (Dec). HRMS: (ASAP): *m*/*z* = 783.7400 [M + H]^+^ (calc. for C_21_H_13_N_3_I_3_S_3_: 783.7403). ^1^H-NMR (300 MHz, DMSO-*d*_6_): δ = 7.85 (d, *J* = 8.5 Hz, 6H, *H*_Ar_), 7.18 (d, *J* = 8.5 Hz, 6H, *H*_Ar_). IR (ATR, cm^−1^): ῡ = 1338 (C–N, vs), 1254 (C–N, s). Raman (neat, cm^−1^): ῡ = 1579 (C=C, s), 1332 (C–N, vs), 1220 (C–H), 440 (C=S, vs).

*1,3,5-Triphenyl-1,3,5-triazinane-2,4,6-trithione* (**4-H**). Following the general procedure with 1,3,5-triphenyl-1,3,5-triazinane-2,4,6-trione (1-H; 103 mg), the title compound was isolated as a yellow solid (116 mg). Yield: 99%. MP: 290–315 °C (Dec). HRMS: (ASAP): *m*/*z* = 406.0501 [M + H]^+^ (calc. for C_21_H_16_N_3_S_3_: 406.0501). ^1^H-NMR (300 MHz, CD_2_Cl_2_) δ = 7.61–7.41 (m, 9H), 7.41–7.31 (m, 6H). ^13^C{^1^H}-NMR (75 MHz, CD_2_Cl_2_) δ = 172.8 (*C*=S), 143.9 (N*C*_Ar_), 129.7 (2*CH*_Ar_), 128.8 (*C*H_Ar_), 128.4 (2*C*H_Ar_). IR (ATR, cm^−1^): ῡ = 1343 (C-N, vs), 1247 (C–N, s). Raman (neat, cm^−1^): ῡ = 1592 (C=C, vs), 1328 (C–N, vs), 1213 (C–H), 439 (C=S, vs).

*1,3,5-Tris(4-tolyl)-1,3,5-triazinane-2,4,6-trithione* (**4-Me**). Following the general procedure with 1,3,5-tris(4-tolyl)-1,3,5-triazinane-2,4,6-trione (1-Me, 112 mg), the title compound was isolated as a yellow solid (125 mg). Yield: 99%. Crystals of the title compound were obtained by slow evaporation of a CHCl_3_ solution of this compound. MP: 300–325 °C (Dec). HRMS: (ASAP): *m*/*z* = 448.0968 [M + H]^+^ (calc. for C_24_H_22_N_3_S_3_: 448.0970). ^1^H-NMR (300 MHz, CD_2_Cl_2_) δ = 7.34 (d, *J* = 8.5 Hz, 6H), 7.21 (d, *J* = 8.5 Hz, 6H), 2.44 (s, 9H). ^13^C{^1^H}-NMR (75 MHz, CD_2_Cl_2_) δ = 173.0 (*C*=S), 141.6 (N*C*_Ar_), 139.1 (*C*_Ar_), 130.3 (*C*H_Ar_), 128.0 (*C*H_Ar_), 21.0 (*C*H_3_). IR (ATR, cm^−1^): ῡ = 1351 (C–N, vs), 1251 (C–N, s). Raman (neat, cm^−1^): ῡ = 1602 (C=C, s), 1328 (C–N, vs), 1202 (C–H, s), 436 (C=S, s).

### 3.3. Isolation of the Mono-Thioisocyanurate Derivative ***6-Me*** and of the Di-thioisocyanurate Derivative ***7-Me***

1,3,5-Tris(4-tolyl)-1,3,5-triazinane-2,4,6-trione (**1-Me**; 1.01 mmol; 403 mg), P_4_S_10_ (0.22 mmol; 100 mg), hexamethyldisiloxane (1.64 mmol; 350 μL), and xylene (5 mL) were added to a Schlenk flask. The mixture was heated at reflux for four days. After cooling to room temperature, the mixture was passed through silica and the solvent was removed under vacuum. The desired mono-, bis-, and tris-thioisocyanurate derivatives were then separated by column chromatography on silica gel (cyclohexane/AcOEt [90:10]).

*6-Thioxo-1,3,5-tris(4-tolyl)-1,3,5-triazinane-2,4-dione* (**6-Me**). The title compound was isolated as a yellow solid (82 mg). Yield: 20%. Crystals of the title compound were obtained by slow evaporation of a CHCl_3_ solution of this compound. HRMS: (ASAP): *m*/*z* = 416.1427 [M + H]^+^ (calc. for C_24_H_22_N_3_O_2_S: 416.1427). ^1^H-NMR (300 MHz, CD_2_Cl_2_) δ = 7.40–7.33 (m, 6H), 7.33–7.20 (m, 7H), 2.46 (s, 9H). ^13^C{^1^H}-NMR (75 MHz, CD_2_Cl_2_) δ = 179.0 (*C*=S), 147.5 (*C*=O), 139.8 (*C*_Ar_), 139.4 (*C*_Ar_), 136.2 (*C*_Ar_), 131.2 (*C*_Ar_), 130.2 (*C*H_Ar_), 130.1 (*C*H_Ar_), 128.2 (*C*H_Ar_), 127.9 (*C*H_Ar_), 21.0 (*C*H_3_), 21.0 (*C*H_3_). IR (ATR, cm^−1^): ῡ = 1709 (C–O, s), 1381 (C–N, vs), 1270 (C–N, s). Raman (neat, cm^−1^): ῡ = 1755 (C–O, s), 1605 (C=C, s), 1315 (C–N, vs), 1211 (C–H).

*4,6-Dithioxo-1,3,5-tris(4-tolyl)-1,3,5-triazinane-2-one* (**7-Me**). The title compound was isolated as a yellow solid (105 mg). Yield: 24%. HRMS: (ASAP): *m*/*z* = 432.1201 [M + H]^+^ (calc. for C_24_H_22_N_3_OS_2_: 432.1199). ^1^H-NMR (300 MHz, CD_2_Cl_2_) δ = 7.41–7.34 (m, 6H), 7.30–7.21 (m, 6H), 2.47 (s, 9H).^13^C{^1^H}-NMR (75 MHz, CD_2_Cl_2_) δ = 176.8 (*C*=S), 145.0 (*C*=O), 141.4 (*C*_Ar_), 139.5 (*C*_Ar_), 139.0 (*C*_Ar_), 136.2 (*C*_Ar_), 130.3 (*C*H_Ar_), 130.2 (*C*H_Ar_), 128.3 (*C*H_Ar_), 127.9 (*C*H_Ar_), 21.1 (*C*H_3_), 21.1 (*C*H_3_). IR (ATR, cm^−1^): ῡ = 1729 (C–O, s), 1367 (C–N, vs), 1268 (C–N, s). Raman (neat, cm^−1^): ῡ = 1729 (C–O, s), 1603 (C=C, s), 1316 (C–N, vs).

*1,3,5-Tris(4-tolyl)-1,3,5-triazinane-2,4,6-trithione* (**4-Me**). This compound was also isolated by chromatography (26 mg). Yield: 6%. See above for the characterization of this compound.

### 3.4. Synthesis of 1,3,5-Tris(4-((9,9-dibutyl-9H-fluoren-2-yl)ethynyl)-phenyl)-1,3,5-triazinane-2,4,6-trithione *(**5**)*

In an oven-dried Schlenk tube, an excess of 9,9-dibutyl-2-ethynyl-9*H*-fluorene (**7**; 178 mg, 0.589 mmol, 5 eq.) was added to 1,3,5-tris(4-iodophenyl)-1,3,5-triazinane-2,4,6-trithione (**4-I**; 90 mg, 0.115 mmol), CuI (0.017 mmol, 3.3 mg, 15 mol.%) and PdCl_2_(PPh_3_)_2_ (0.008 mmol, 5.6 mg, 7 mol.%) in a mixture of DMF/Et_3_N (2:3). After seven days of stirring at 70 °C, the reaction medium was allowed to cool to room temperature and the reaction mixture was diluted in CH_2_Cl_2_ (20 mL) washed with water (3 × 50 mL) and dried over MgSO_4_. After filtration and evaporation to dryness, the crude product was purified by column chromatography on silica gel using a mixture of petroleum ether/CH_2_Cl_2_ (8:2) as the eluent. After recrystallization from pentane, the title compound was isolated as a yellow powder (16 mg). Yield 11%. *R*_f_: 0.42 (petroleum ether/CH_2_Cl_2_ [8:2]). HRMS: (ESI): *m*/*z* = 1306.6134 [M + H]^+^ (calc. for C_90_H_88_N_3_S_3_: 1306.6135). Anal. Calc. for C_90_H_87_N_3_S_3_: C, 82.71, H, 6.71, N, 3.22, S, 7.36; found: C, 82.40, H, 6.68, N, 3.13, S, 6.88. ^1^H-NMR (300 MHz, CDCl_3_): δ = 7.80–7.66 (m, 4H, *H*_Ar_), 7.53 (m, 2H, *H*_Ar_), 7.42–7.31 (m, 5H, *H*_Ar_), 2.00 (m, *J* = 8.2 Hz, 4H, C*H*_2Bu_), 1.11 (h, *J* = 7.4 Hz, 4H, C*H*_2Bu_), 0.70 (t, *J* = 7.4 Hz, 6H, C*H*_3Bu_), 0.67–0.51 (m, 4H, C*H*_2Bu_). ^13^C{^1^H}-NMR (75 MHz, CDCl_3_): δ = 172.8, 151.8, 151.5, 143.4, 142.5, 141.0, 133.7, 131.4, 129.2, 128.3, 127.6, 126.7, 125.2, 123.6, 121.7, 120.7, 120.3, 93.0, 89.1, 55.8, 40.8, 26.6, 23.7, 14.5. IR (ATR, cm^−1^): ῡ = 2201 (C≡C, vs), 1349 (C–N, vs), 1257 (C–N, s). Raman (neat, cm^−1^): ῡ = 2198 (C≡C, vs), 1596 (C=C, vs), 1334 (C–N, vs), 1224 (C–H), 428 (C=S, vw).

### 3.5. Spectroscopic Measurements

All photophysical measurements were performed with freshly prepared air-equilibrated THF solutions (HPLC grade) at room temperature (298 K). UV–Vis absorption spectra were recorded on a Jasco V-570 spectrophotometer (Mary’s Court Easton, MD, USA). The samples used to make the solutions were freshly recrystallized or thoroughly washed with cooled ether/pentane prior to the measurements to remove any organic impurity. Steady state fluorescence studies were performed in dilute air-equilibrated solutions in quartz cells of 1 cm path length (ca. 1 × 10^−6^ M, optical density < 0.1) at room temperature (20 °C) using an Edinburgh Instruments (FLS920) spectrometer (Edinburgh, UK) in photon-counting mode. Fully corrected excitation and emission spectra were obtained with an optical density at *λ*_exc_ ≤ 0.1. Fluorescence quantum yields were measured according to procedures in the literature [41,42]. UV-vis absorption spectra used for the calculation of the fluorescence quantum yields were recorded using a double-beam Jasco V-570 spectrometer.

### 3.6. X-ray Crystallography

Data collection was undertaken on monocrystals of **4-Me** and **6-Me** mounted with a cryoloop on the goniometer head of a D8 VENTURE Bruker AXS diffractometer equipped with a (CMOS) PHOTON 100 detector using Mo-K radiation (*λ* = 0.71073 Å, multilayer monochromator) at T = 150 K. The structures were solved by a dual-space algorithm using the SHELXT program, [43] and then refined with full-matrix least-squares methods based on *F^2^* (SHELXL program [44]). All non-hydrogen atoms were refined with anisotropic atomic displacement parameters. H atoms were finally included in their calculated positions in the final cycle of refinement, treated as riding on their parent atom with constrained thermal parameters. Refinement on *F*^2^ with, respectively, 10,200 or 4732 unique intensities and 547 or 281 parameters converged at *ω_R_*(*F*^2^) = 0.1223 (*R_F_* = 0.0485) or 0.1658 (*R_F_* = 0.0931) for 8174 or 3498 observed reflections with *I >* 2*σ*(*I*). The data have been deposited at the Cambridge Crystallographic Data Center (CCDC 2042234 and CCDC 2042235, respectively) [45].

### 3.7. DFT Computations

The DFT calculations reported in this work were performed using the Gaussian09 [46] program. The geometries of all compounds were optimized without symmetry constraints using the PBE1PBE-GD3BJ functional [47] and the 6-31G* basis set. The solvent effects were taken into account by means of the polarizable continuum model (PCM). [48] Calculations of the normal modes of vibration were carried out to confirm the true minima character of the optimized geometries. TD-DFT calculations were performed at the same level of theory using the previously optimized geometries. Swizard [49] was used to plot the simulated spectra and GausView [50] was used for the MO plots.

## 4. Conclusions

Five triaryl-1,3,5-triazinane-2,4,6-trithiones (or *N*,*N*’,*N*”-triarylthioisocyanurates) were synthesized and characterized in this work. **4-I** and **4-Br** are key synthetic precursors that can be used to access extended isocyanurates such as **5** by cross-coupling reactions. Thioisocyanurates have been previously mentioned in the literature, but to the best of our knowledge, no characterization of any example has been reported so far, apart from the x-ray structure of **4-H** [13]. The additional characterization reported for **4-X** (X = Br, I, H, Me) and **5** should facilitate the straightforward experimental identification of such compounds. For instance, given that the carbonyl stretching motion provides a simple and powerful means to characterize isocyanurates [3], we looked for the vibrational signature of the ν(C=S) modes in thioisocyanurates using a combined Raman-IR approach, backed by DFT calculations. A symmetric breathing motion of the heterocycle involving the C=S stretch was detected as a strongly Raman-active mode near 440 cm^−1^ for all derivatives **4-X** and **5**. Other intense IR-active ν(C–N) modes are also proposed as diagnostic vibrational markers for these thioisocyanurate derivatives. Finally, it is worth emphasizing that the structural data obtained for **4-Me** in the solid state were consistent with the structural data previously reported for **4-H**. It confirmed the existence of electron delocalization within the central heterocycle, a feature also supported by DFT calculations which has been previously reported for isocyanurates [3].

We also report the synthesis and characterization of the mono- and bis-thionated derivatives **6-Me** and **7-Me**. Along with **4-Me**, these new compounds allowed us to experimentally monitor the impact of the stepwise replacement of oxygen by sulfur on the molecular properties starting from the known isocyanurate **1-Me**. Our work indicates that introduction of sulfur progressively reduces the HOMO-LUMO gap proceeding from **1-Me** to **4-Me**, resulting in a red shift of the main absorption band of these molecules. The latter is located in the UV range and corresponds to a π→π* transition with a CT character from the peripheral arms toward the central heterocycle. Increasing the number of thiocarbonyl moieties at the central heterocycle also induces the presence at low energies of a set of symmetry-forbidden (very weakly allowed) states, resulting in efficient quenching of the fluorescence in thioisocyanurates. This is seen even with derivatives featuring extended arms terminated by good fluorophores such as **5**. Thus, while the reduction of the molecular bandgap seen for thioisocyanurates and the increased polarizability of sulfur over oxygen are important assets in the development of more NLO-active molecules, the non-emissive nature of these compounds precludes the determination of their 2PA cross-sections by two-photon excited fluorescence (TPEF). Z-scan investigations are now required to unravel the nonlinear absorption properties of these promising octupoles.

## Figures and Tables

**Table 1:** Characteristic spectral signatures of the new thiosocyanurates.

Cmpd.	υ_(C≡C)_ (cm^−1^)	υ _(C=X)_ (X = O, S) (cm^−1^)	δ_(C≡C)_^a^ (ppm)	δ_(C=X)_^a^ (ppm)
**1-Me ^b^**	/	1772 (s) ^c^, 1694 (vs) ^d,e,f^	/	148.8
**3**	2203 ^c,d^	1777 (s) ^c^, 1720 (vs) ^d^	92.8, 88.9	148.4
**4-Br**	/	438 (vs) ^d^	/	/
**4-I**	/	440 (vs) ^d^	/	/
**4-H**	/	439 (vs) ^d,g^	/	172.8 ^h^
**4-Me**	/	436 (s) ^d,g^	/	172.8
**5**	2198 ^c,d^	428 (vw) ^d^	93.0, 89.1	172.8
**6-Me**	/	1755 (s) ^c,d,g^, 1709 (s) ^c^	/	178.8, 147.5
**7-Me**	/	1728 (s) ^c,d,g^	/	176.5, 145.1

^a 13^C{^1^H}-NMR in CDCl_3_ unless specified. ^b^ Data from [3,30]. ^c^ FTIR in ATR (±4 cm^−1^). ^d^ Raman spectrum on a solid sample with excitation at 633 nm (±2 cm^−1^). ^e^ Data from [4]. ^f^ Fermi coupling (second band at 1720 cm^−1^). ^g^ Fluorescence observed from sample during Raman spectrum acquisition. ^h^ In CDCl_3_.

**Table 2:** Photophysical data for the new thioisocyanurates and those for previously reported isocyanurate analogues at 25 °C.

Cmpd.	*λ* ^max^_abs4_	*ε*_max4_	*λ* ^max^_abs3_	*ε*_max3_	*λ* ^max^_abs2_	*ε*_max2_	*λ* ^max^_abs1_	*ε*_max1_	*λ* ^max^_em_	*Φ*_F_^a^	Stokes Shift ^b^	DFT ^c,d^	Ref.
	(*λ*^max^ in nm and *ε*_max_ in 1 × 10^3^ cm^−1^ M^−1^)								(nm)		(cm^−1^)	*λ*^max^ (nm) [*f* ]	
**1-Br**	/	/	/	/	<240	/	262 (sh)	1.5	306	0.0006	5200	nc	This work and [3]
**1-I**	/	/	/	/	<240	/	266 (sh)	6.3	/	0.0	/	nc	[39]
**1-H**	/	/	/	/	251	74.5	256 (sh)	0.8	283	0.005	3730	nc	[3]
**1-Me**	/	/	/	/	<240	/	261 (sh)	1.0	290	0.004	3830	207 [0.30] 207 [0.30]	This work and [3]
**3**	/	/	/	/	327	101	344	101	355	0.60	900	345 [2.83] ^e^ 345 [2.81] ^e^	This work and [4]
**4-Br**	<240	/	297	51.7	378 (sh)	0.3	468	0.1	/	0.0	/	nc	This work
**4-I**	<240	/	295	48.8	380 (sh)	0.2	467	0.1	/	0.0	/	nc	This work
**4-H**	<240	/	300	48.1	378 (sh)	0.3	468	0.1	/	0.0	/	nc	This work
**4-Me**	260 (sh)	12.3	300	32.4	380 (sh)	0.2	475	0.05	/	0.0	/	279 [0.40] 279 [0.40]	This work
**5**	331	177	351	167	438	0.3	470	0.3	/	0.0	/	367 [2.36] 367 [2.37]	This work
**6-Me**	278	15.1	304 (sh)	8.8	380	0.1	440 (sh)	0.02	/	0.0	/	252 [0.16]	This work
**7-Me**	252	16.5	304	24.7	381	0.1	440	0.06	/	0.0	/	279 [0.35]	This work

^a^ Fluorescence quantum yield (±10%) in CH_2_Cl_2_ when excited at *λ*_abs_ (standard: quinine bisulfate in H_2_SO_4_ 0.5 M). ^b^ Stokes shift = (1/*λ*^max^_abs_ − 1/*λ*^max^_em_). ^c^ First allowed transition (*f* > 0.1) calculated at the PBE1PBE-GD3BJ/6-31G* level on (computationally-simpler) model complexes in CH_2_Cl_2_ (*f* = oscillator strength). ^d^ nc: not computed. ^e^ Calculated for a computationally-simpler model complex of **3** at the MPW1PW1/6-31G* level in CH_2_Cl_2_ [4].

**Table 3:** Calculated dipole moments and HOMO-LUMO gaps in CH_2_Cl_2_ for selected derivatives (C1 symmetry) in their most abundant GS conformation (C1).

Cmpds.	μ (D)	HOMO-LUMO Gap (eV)	Ref.
**1-Me**	0.10	6.62	This work
**3′ ^a^**	0.77	4.17	This work
**4-Me**	0.09	3.87	This work
**5′**	0.73	3.61	This work
**6-Me**	1.62	4.86	This work
**7-Me**	1.40	4.15	This work

^a^ See also [4] for values computed for another model complex of **3** at the PBE1PBE-GD3BJ/6-31G* level in CH_2_Cl_2_ (PCM).

## Supplementary Materials

The following are available online, ^1^H-NMR and ^13^C-NMR of the new compounds, selected crystallographic, vibrational (IR and Raman), solvatochromy and electronic emission data, and selected DFT data (Cartesian coordinates, optimized GS geometries, FMOs, dipole moments, vibrational properties, TD-DFT outputs, first triplet states).
