# Supplementary material for: 1,3,5-Triaryl-1,3,5-Triazinane-2,4,6-Trithiones: Synthesis, Electronic Structure and Linear Optical Properties
Source: Molecules. 2020 Nov 23;25(22):5475. doi: 10.3390/molecules25225475 (PMC7700228; doi:10.3390/molecules25225475)
Supplement: Supplementary file 1 [file molecules-25-05475-s001.pdf]

Supporting Information

1,3,5-Triaryl-1,3,5-Triazinane-2,4,6-Trithiones: Synthesis, Electronic Structure and Linear Optical Properties

Ismaël Rabouel,[a] Nicolas Richy,[a] Anissa Amar,[b,c] Abdou Boucekkine,[a,*] Thierry Roisnel,[a] Olivier Mongin,[a] Mark G. Humphrey[d,*] and Frédéric Paul[a,*]

[a] *Univ Rennes, CNRS, ISCR (Institut des Sciences Chimiques de Rennes), UMR 6226, 35000 Rennes (France)*

[b] *Département de Chimie, Faculté des Sciences, Université Mouloud Mammeri, 15000 Tizi-Ouzou (Algeria)*

[c] *Faculté de Chimie, Université des Sciences et de la Technologie Houari-Boumediene, 16111 Bab-Ezzouar (Algeria)*

[d] *Research School of Chemistry, Australian National University, Canberra, ACT 2601 (Australia)*

Corresponding authors:* frederic.paul@univ-rennes1.fr, abdou.boucekkine@univ-rennes1.fr *and* mark.humphrey@anu.edu.au

Tel: (+33) 02 23 23 59 62 (F.P.)

Including:

1. ^{1}H / $^{13}C\{^{1}H\}$ NMR spectra of selected compounds p. S2
2. X-ray data for **4-Me** and **6-Me** p. S9
3. Infrared and Raman spectra p. S11
4. Solvatochromy of **4-X** and **5** at 298K p. S13
5. Emission data for **1-Me**, **4-Me** and **5** at 77K p. S14
6. Cartesian coordinates of the DFT optimized geometries for **1-Me**, **4-Me**, **5'**, **6-Me** and **7-Me** p. S15
7. Selected frontier MOs for **1-Me**, **4-Me**, **6-Me**, **7-Me** and **5'** p. S20
8. Computed atomic charges and bond orders for **1-Me** and **4-Me** p. S21
9. Computed dipole moments for **1-Me**, **4-Me**, **5'**, **6-Me** and **7-Me** p. S22
10. Computed vibrational spectra for **1-Me**, **4-Me**, **6-Me** and **7-Me** p. S23
11. Computed singlet lowest-lying transitions and energies of the first triplet state computed for **1-Me**, **4-Me**, **5'**, **6-Me** and **7-Me** p. S26
12. Computed energies of the first triplet state for **1-Me**, **3'**, **4-Me, 5'**, **6-Me** and **7-Me** p. S29
13. References p. S29

1. ^{1}H / $^{13}C\{^{1}H\}$ NMR spectra of selected compounds

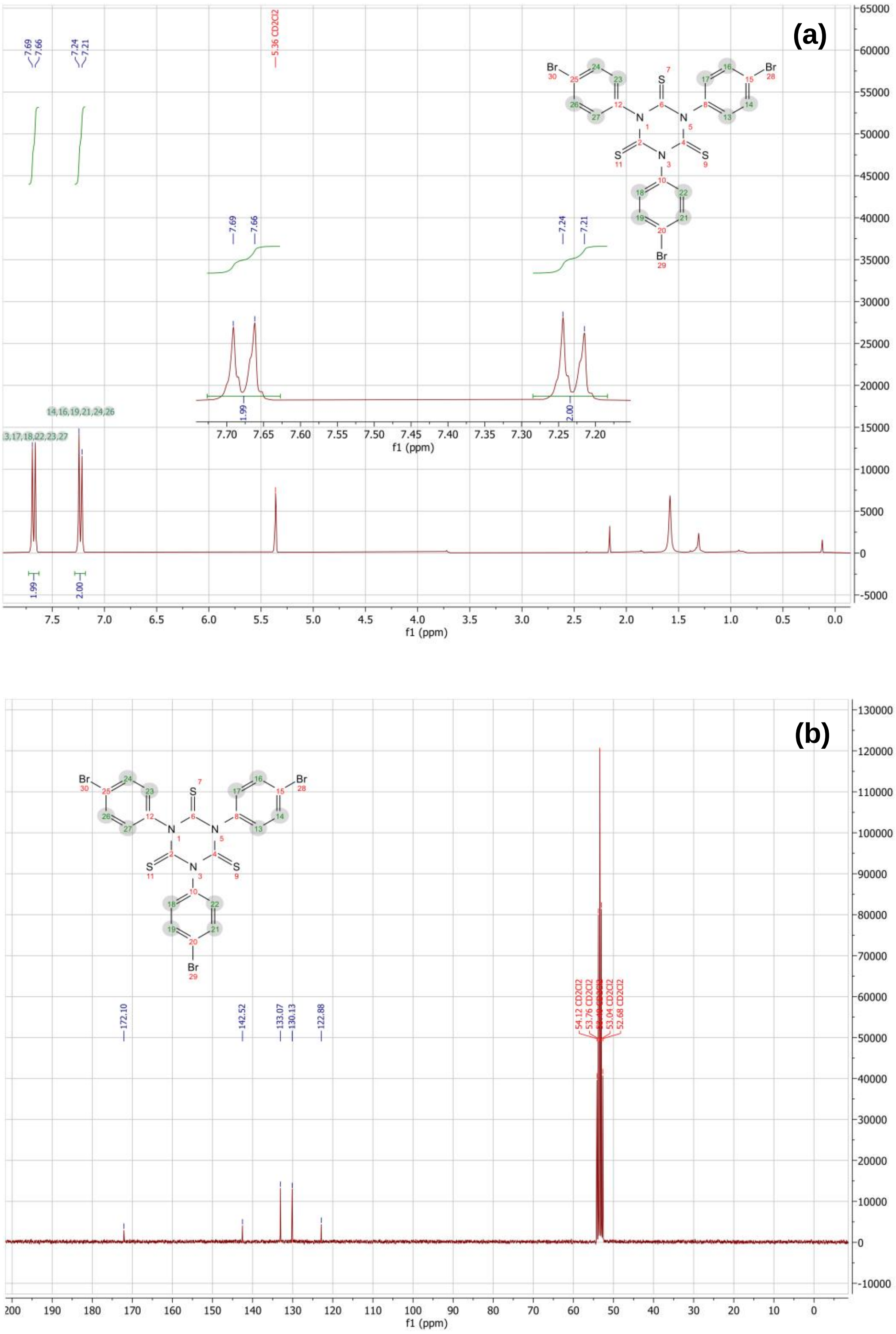

Figure S1. ^{1}H (a) and $^{13}C\{^{1}H\}$ (b) NMR spectra at 300 and 75 MHz, respectively, for **4-Br** in CD_2Cl_2.

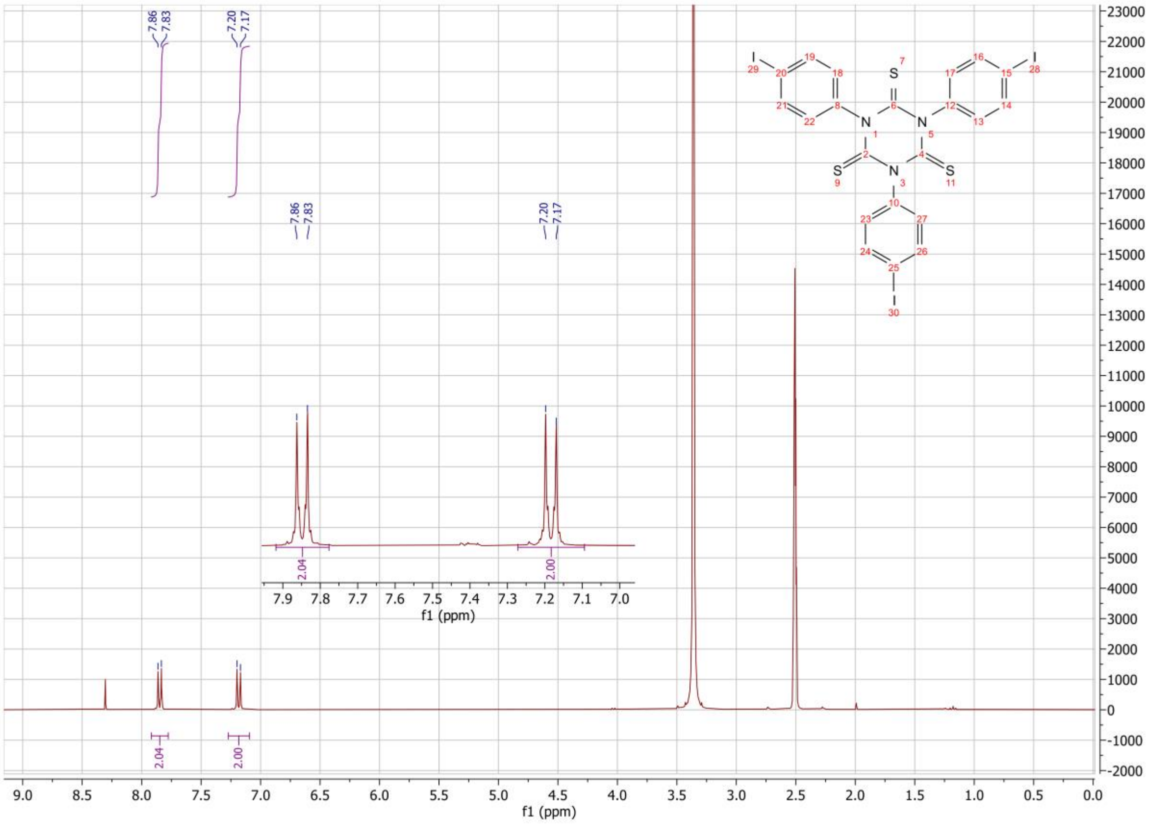

Figure S2. ^{1}H (a) NMR spectrum at 300 MHz for **4-I** in DMSO-d_6.

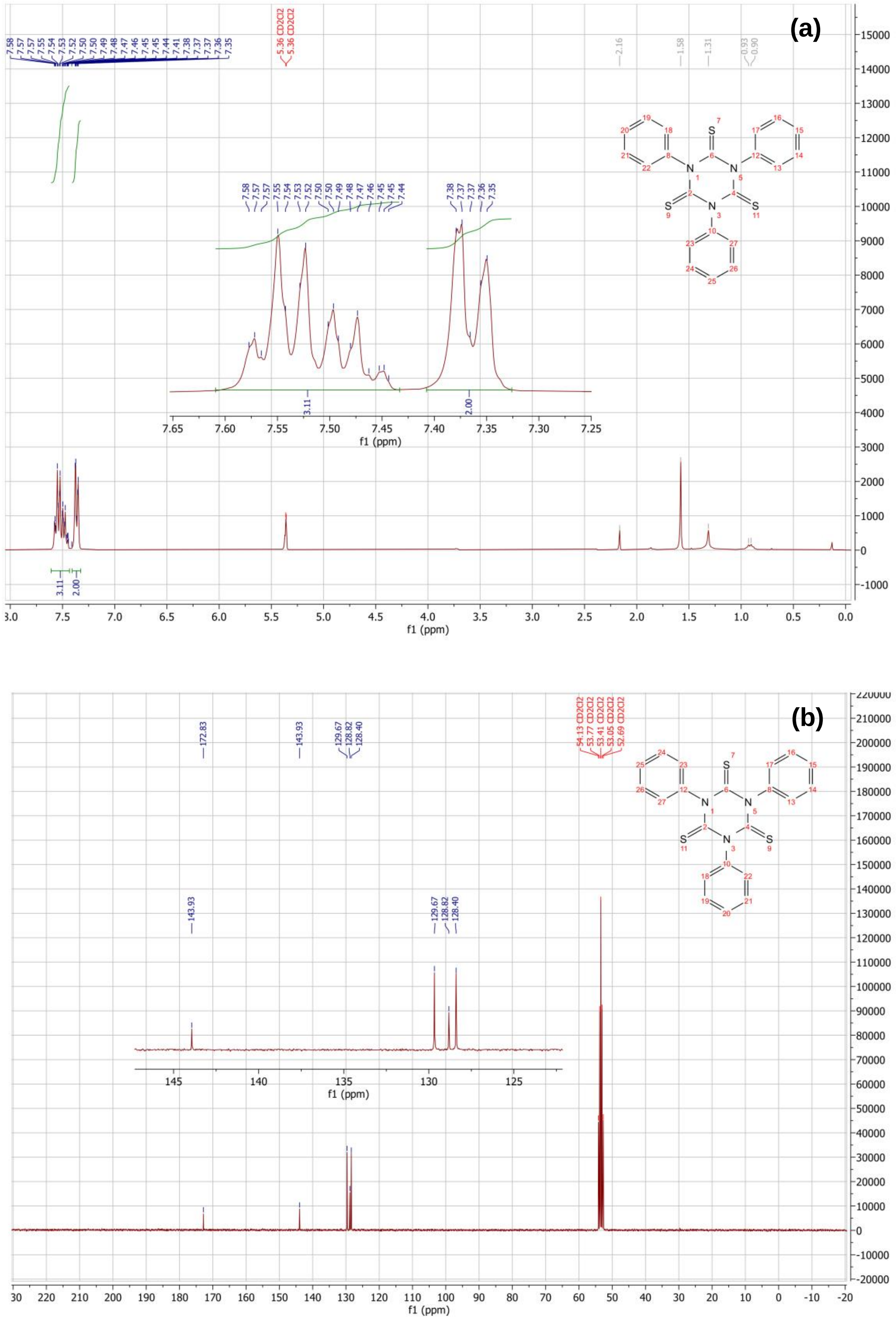

Figure S3. ^{1}H (a) and $^{13}C\{^{1}H\}$ (b) NMR spectra at 300 and 75 MHz, respectively, for **4-H** in CD_2Cl_2.

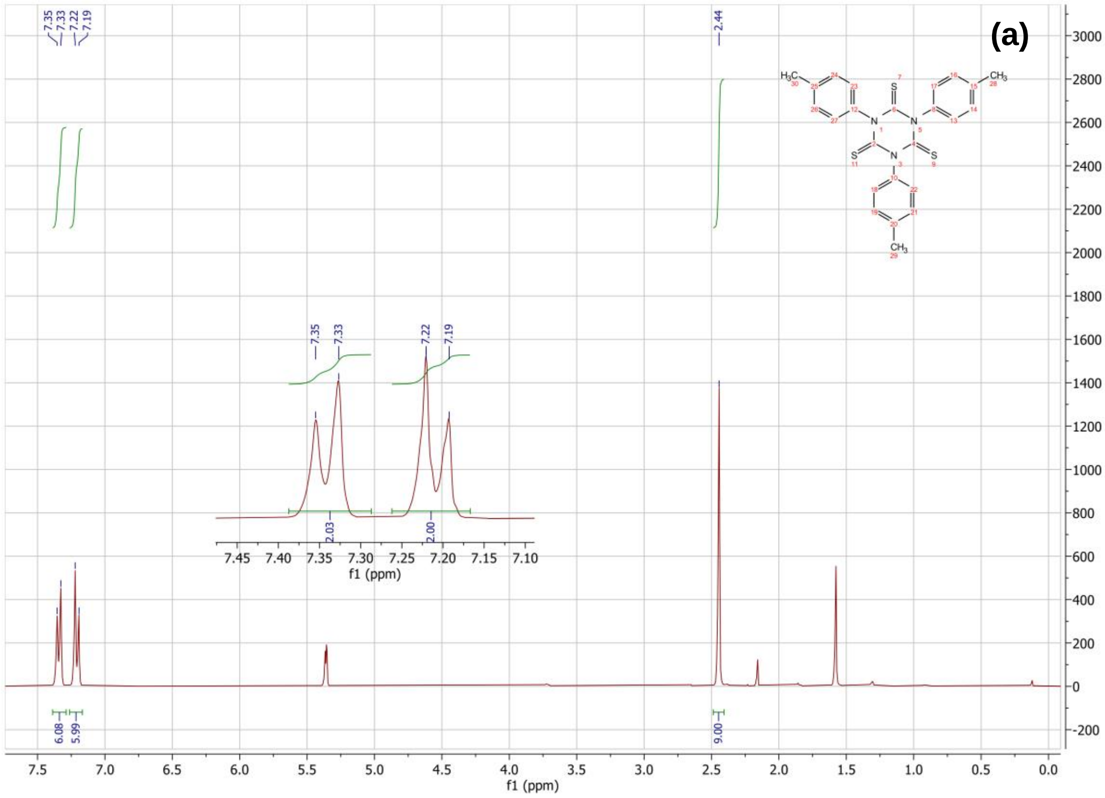

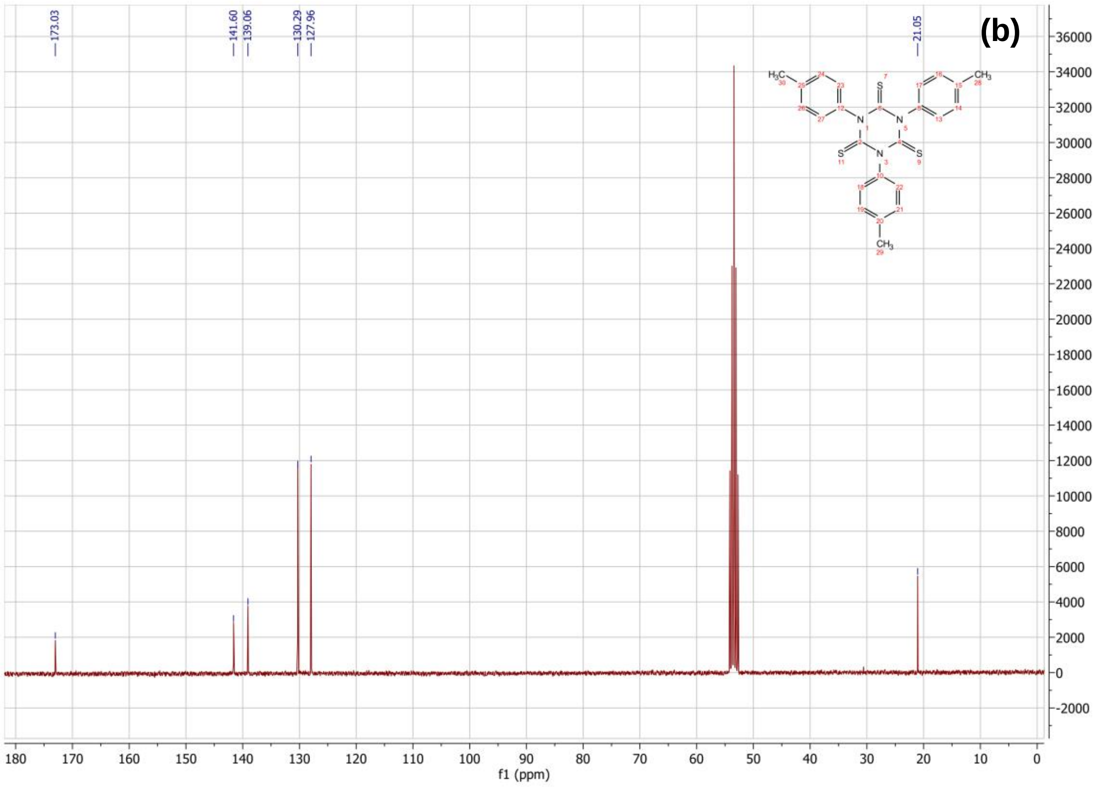

Figure S4. ^{1}H (a) and $^{13}C\{^{1}H\}$ (b) NMR spectra at 300 and 75 MHz, respectively, for **4-Me** in CD_2Cl_2.

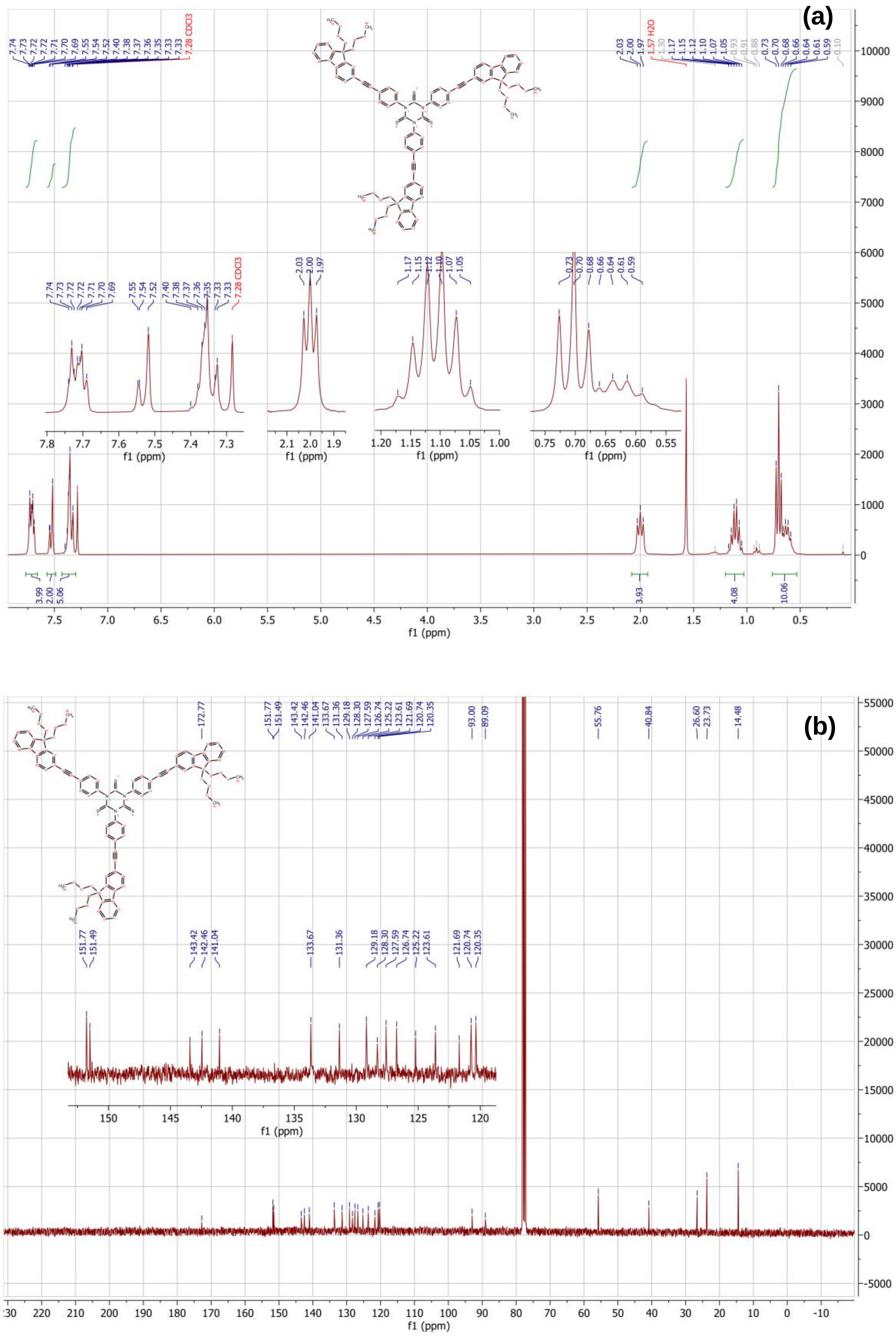

Figure S5. ^{1}H (a) and $^{13}C\{^{1}H\}$ (b) NMR spectra at 300 and 75 MHz, respectively, for **5** in CD_2Cl_2.

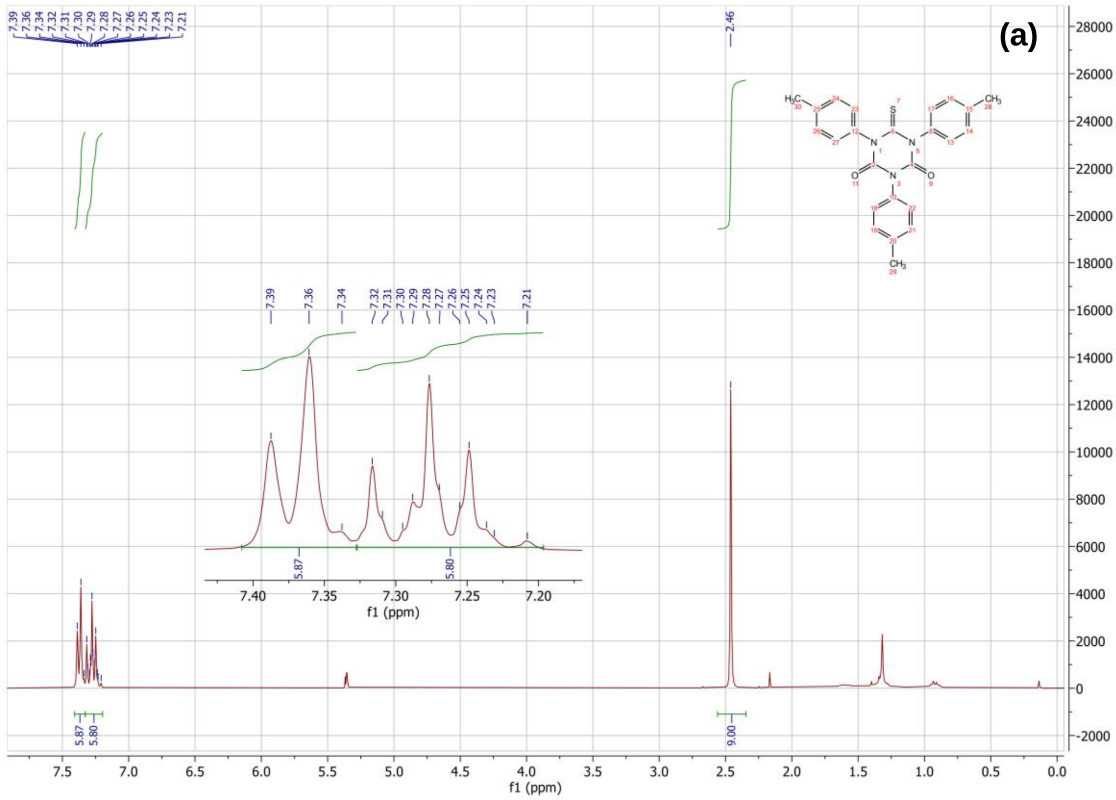

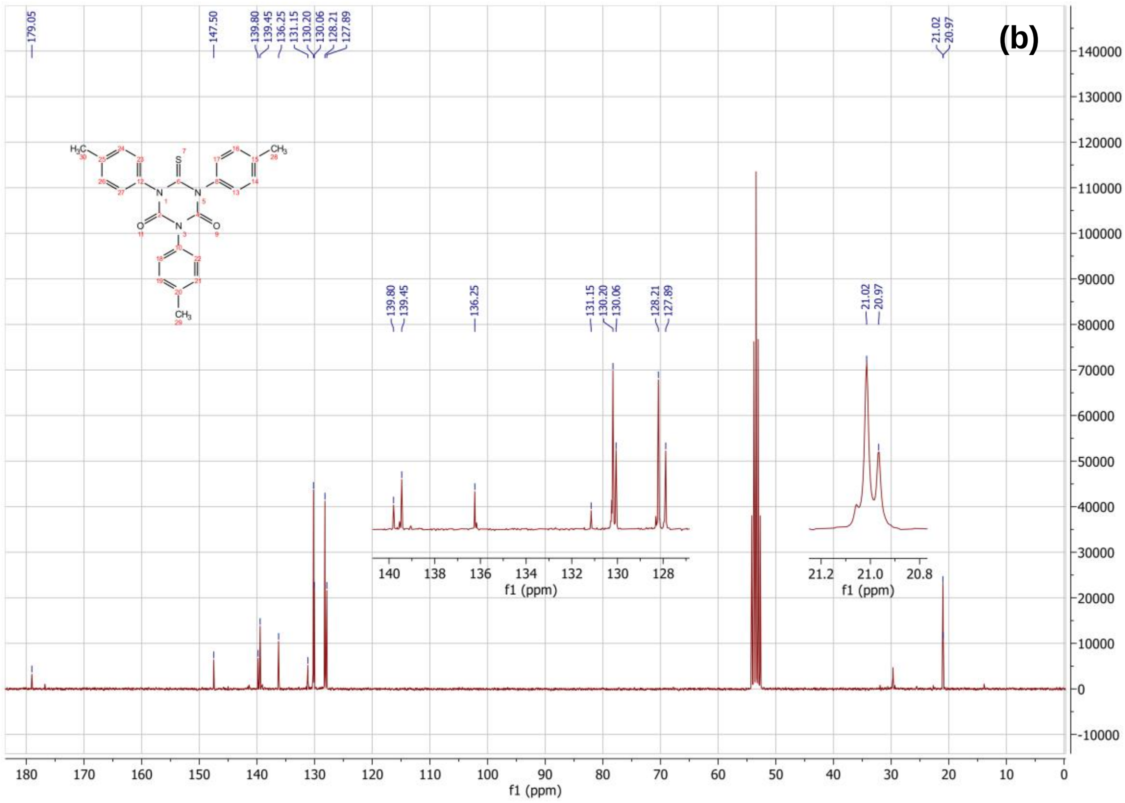

Figure S6. ^{1}H (a) and $^{13}C\{^{1}H\}$ (b) NMR spectra at 300 and 75 MHz, respectively, for **6-Me** in CD_2Cl_2.

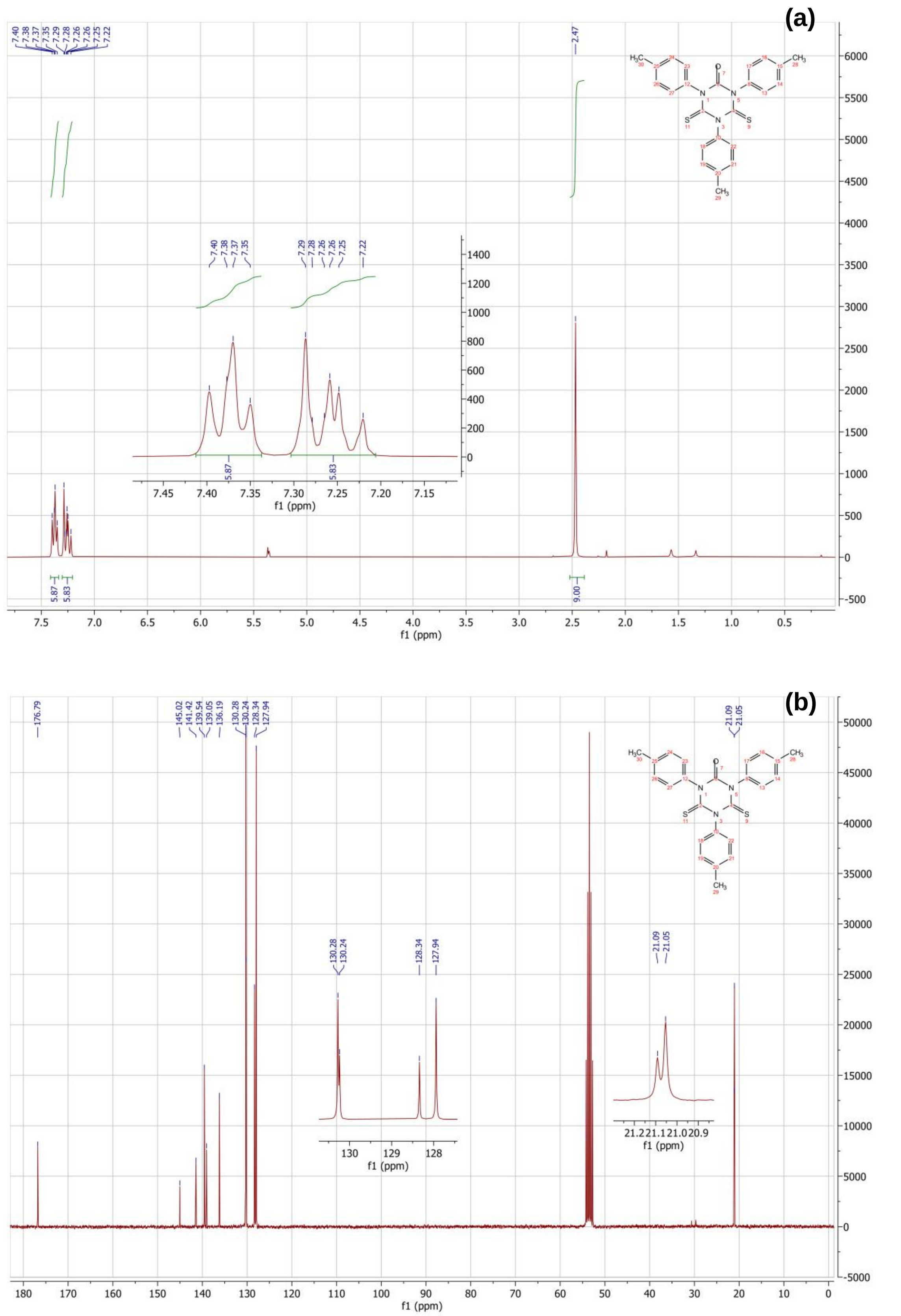

Figure S7. ^{1}H (a) and $^{13}C\{^{1}H\}$ (b) NMR spectra at 300 and 75 MHz, respectively, for **7-Me** in $CDCl_3$.

2. X-ray data for 4-Me and 6-Me

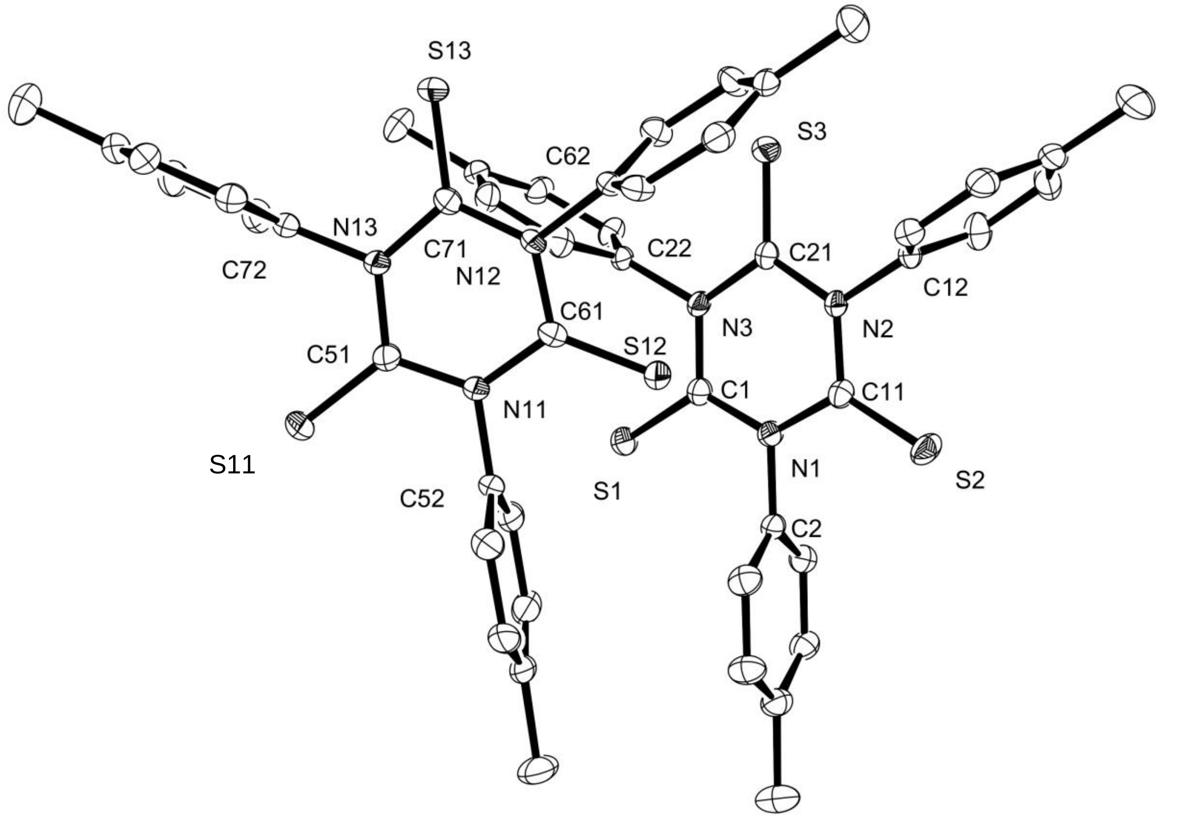

Figure S8. ORTEP representation of the asymmetric unit of **4-Me** in the solid state at the 50 % probability level. S1-C1 1.650(2), S2-C11, 1.637(2), S3-C21 1.637(2), C1-N1 1.384(2), C11-N1 1.391(3), C11-N2 1.388(3), C21-N2 1.390(2), C1-N3 1.385(2), C21-N3 1.396 (2), N1-C2 1.456(2), N2-C12 1.461(2), N3-C22 1.458(2), S11-C51 1.638(2), S12-C61, 1.645(2), S13-C71 1.636(2), C51-N11 1.388(3), C61-N11 1.390(2), C61-N12 1.391(2), C71-N12 1.397(2), C51-N13 1.392(2), C71-N13 1.394(2), N11-C52 1.458(2), N12-C62 1.451(2), N13-C72 1.454(2) Å.

Table S1. Crystal data, data collection, and refinement parameters for **4-Me** and **6-Me**.

Cmpd	4-Me	6-Me
formula	$C_{24}H_{21}N_3S_3$	$C_{24}H_{21}N_3O_2S$
fw (g)	447.62	415.50
cryst. syst.	monoclinic	orthorhombic
space group	$P2_1/n$	P_{bcn}
a (Å)	14.3342(12)	19.4944(10)
b (Å)	19.3756(13)	9.5037(5)
c (Å)	16.7906(12)	22.3666(11)
α (deg)	90.0	90.0
β (deg)	107.008(3)	90.0
γ (deg)	90.0	90.0
V (Å^3)	4459.4(6)	4143.8(4)
Z	8	8
D_{calc} (g cm^{-3})	1.333	1.332
crystal size (mm)	0.58 × 0.32 × 0.09	0.23 × 0.06 × 0.04
F(000)	1872	1744
abs. coef. (mm^{-1})	0.349	0.182
N° total refl. / N° unique refl.	30215/10200	51166/4732
N° of variables/ N° refl. > 2σ(I)	547/10200	281/4732
final R	0.0485	0.0931
R_W	0.1223	0.1658
Goodness of fit / F^2 (S_W)	1.044	1.220

3. Infrared and Raman spectra

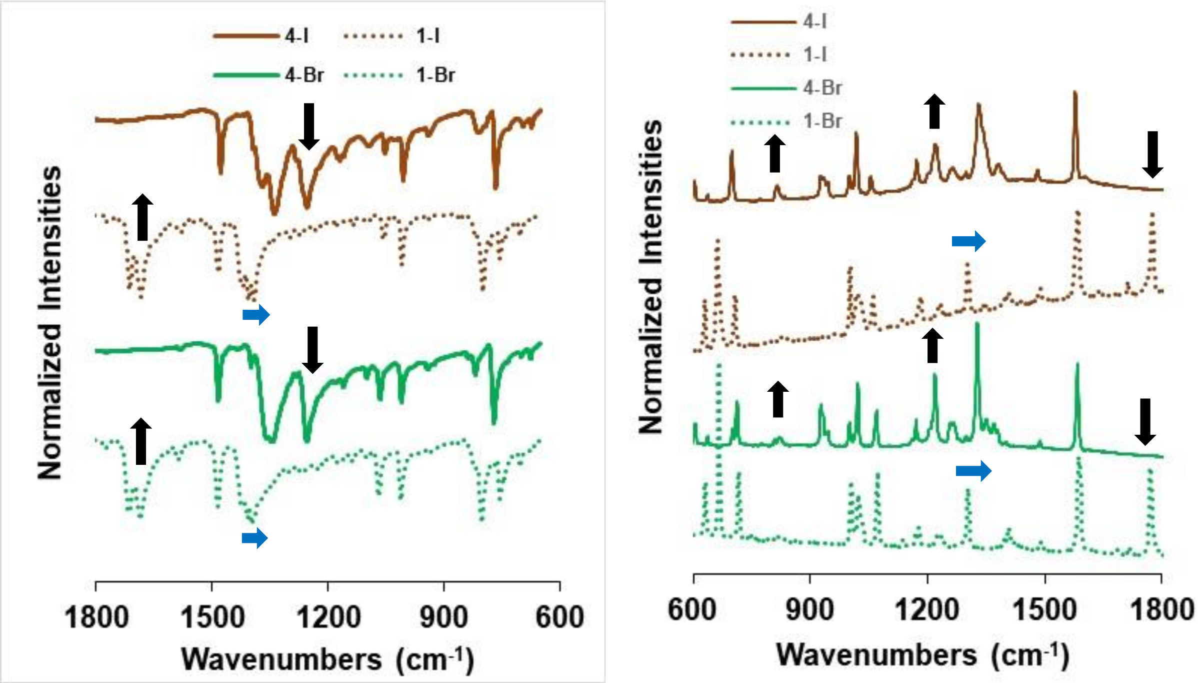

Figure S9. Infrared (ATR) and Raman spectra of selected **1-X** and **4-X** derivatives (X = Br, I) in the solid state at 25 °C showing the change in vibrational modes between the two families of products. Note that ν(C-X) modes (X = Br, I) are most likely located outside the spectral range shown (ν(C-I) are traditionally around 500-600 cm^{-1} and ν(C-Br) around 500-700 cm^{-1}).[1,2]

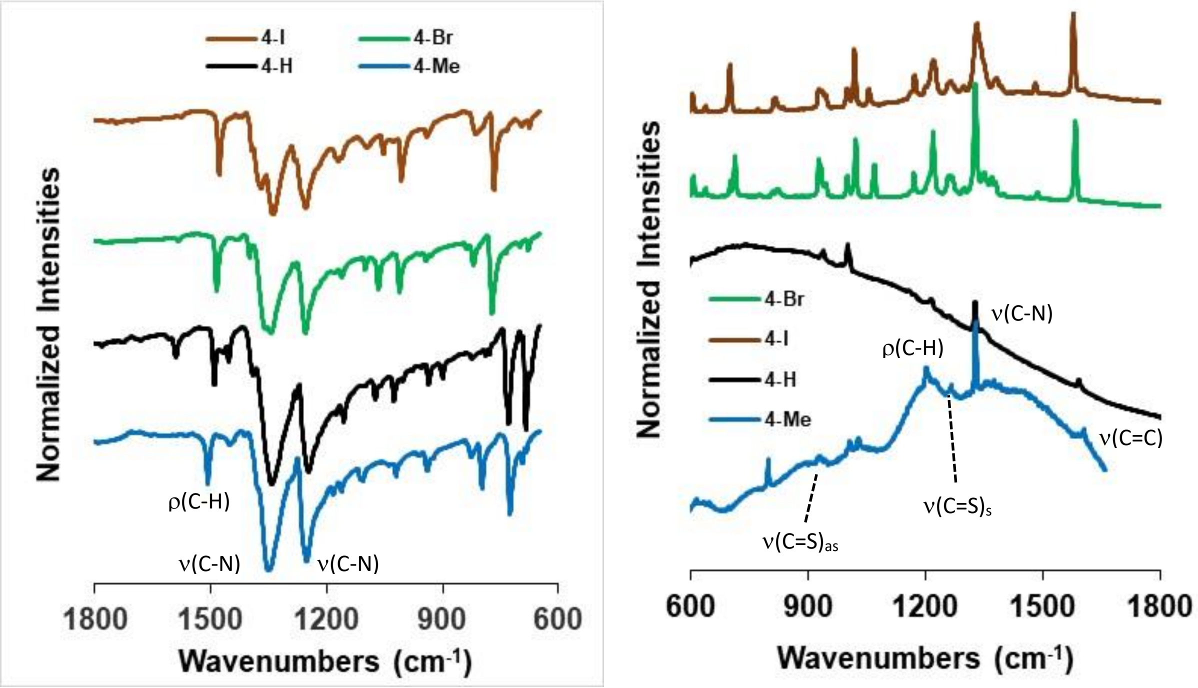

Figure S10. Infrared (ATR) and Raman spectra of **4-X** derivatives (X = Br, I, H, Me) in the solid state at 25 °C. Some of the characteristic vibrational modes are labelled based on DFT calculations on **4-Me**.

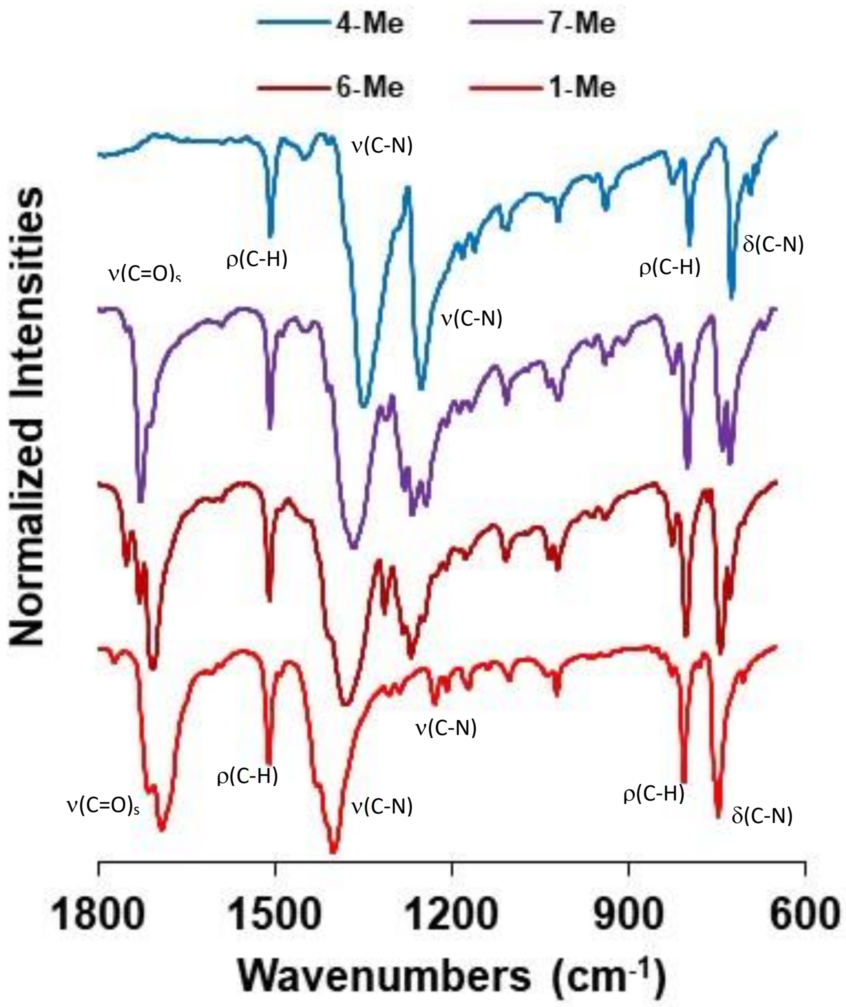

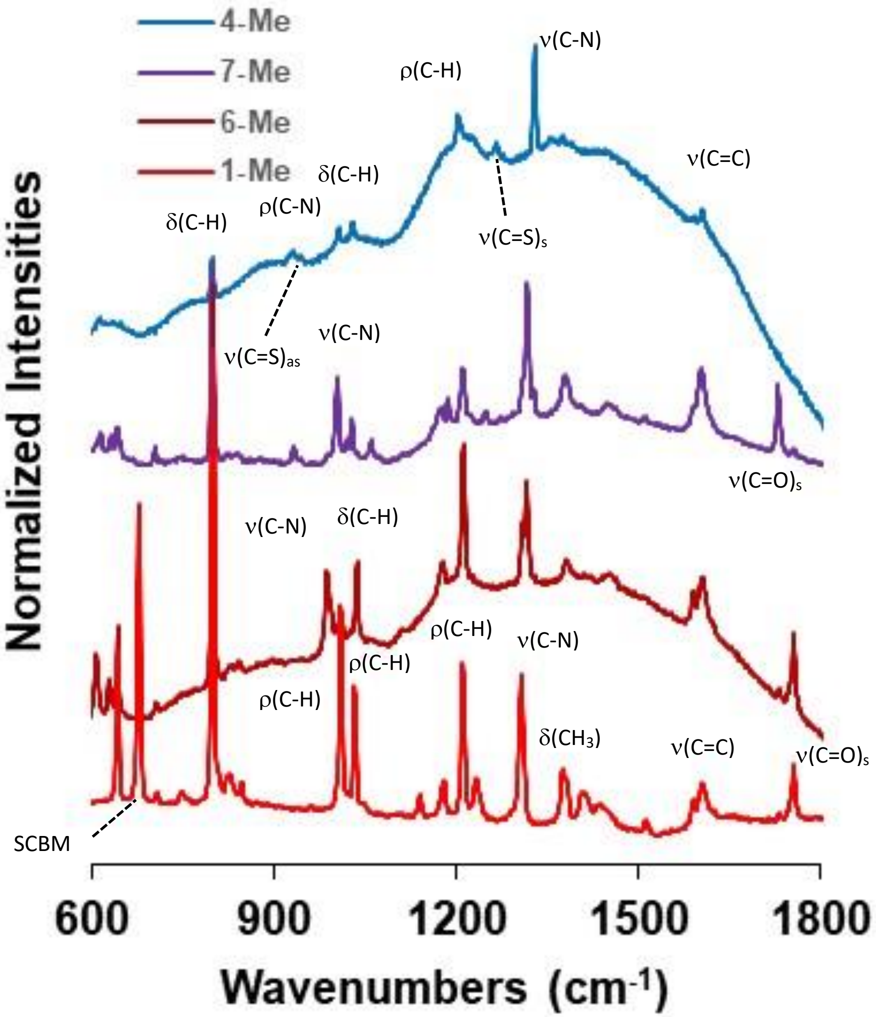

Figure S11. Infrared (ATR) and Raman spectra of **1-Me**, **4-Me**, **6-Me** and **7-Me** derivatives in the solid state at 25. An attribution of the main vibrational modes is proposed based on DFT calculations. The symmetric “breathing mode” of the core coupled with the C=O stretch is indicated as “SCBM”.

4. Solvatochromy of 4-X and 5 at 298K

Table S2. Solvatochromy of **4-X** (X = Br, I, H, Me) and **5** (298K).

	λ^{max}_{abs} (nm)		
**Cmpd\Solvent**	**THF**	**CH_2Cl_2**	**DMF**
**4-Br**	294	297	299
**4-I**	296	295	297
**4-H**	300	300	301
**4-Me**	300	300	301
**5**	329, 350	331, 351	330, 351

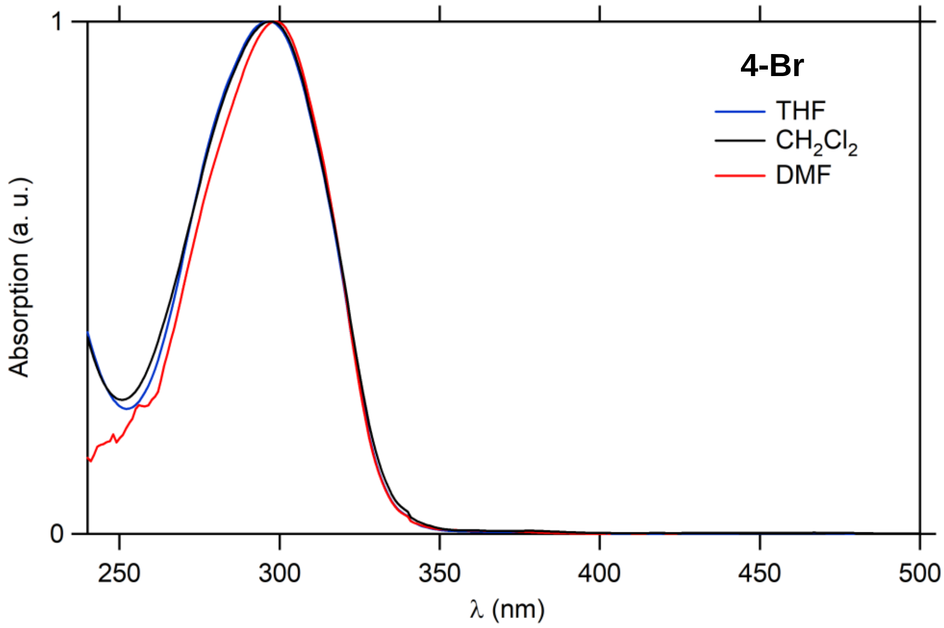

Figure S12. Solvatochromy of **4-Br** at 298K.

5. Emission data for 1-Me, 4-Me and 5 at 77K

Table S3. Emission and lifetime measurements for selected compounds at 77 K.[a]

Cmpd	λ_{abs} (nm)	λ_{em} (nm)	τ (ns)
**1-Me**[b]	259	285, 393	/
**4-Me**	298	489, 516	/
**5**	328	515	< 0.5

[a] Fluorescence quantum yields could not be determined because of their weakness.[3] [b] Possible phosphorescence at λ_{em} = 393 nm, Φ_P << 0.5.

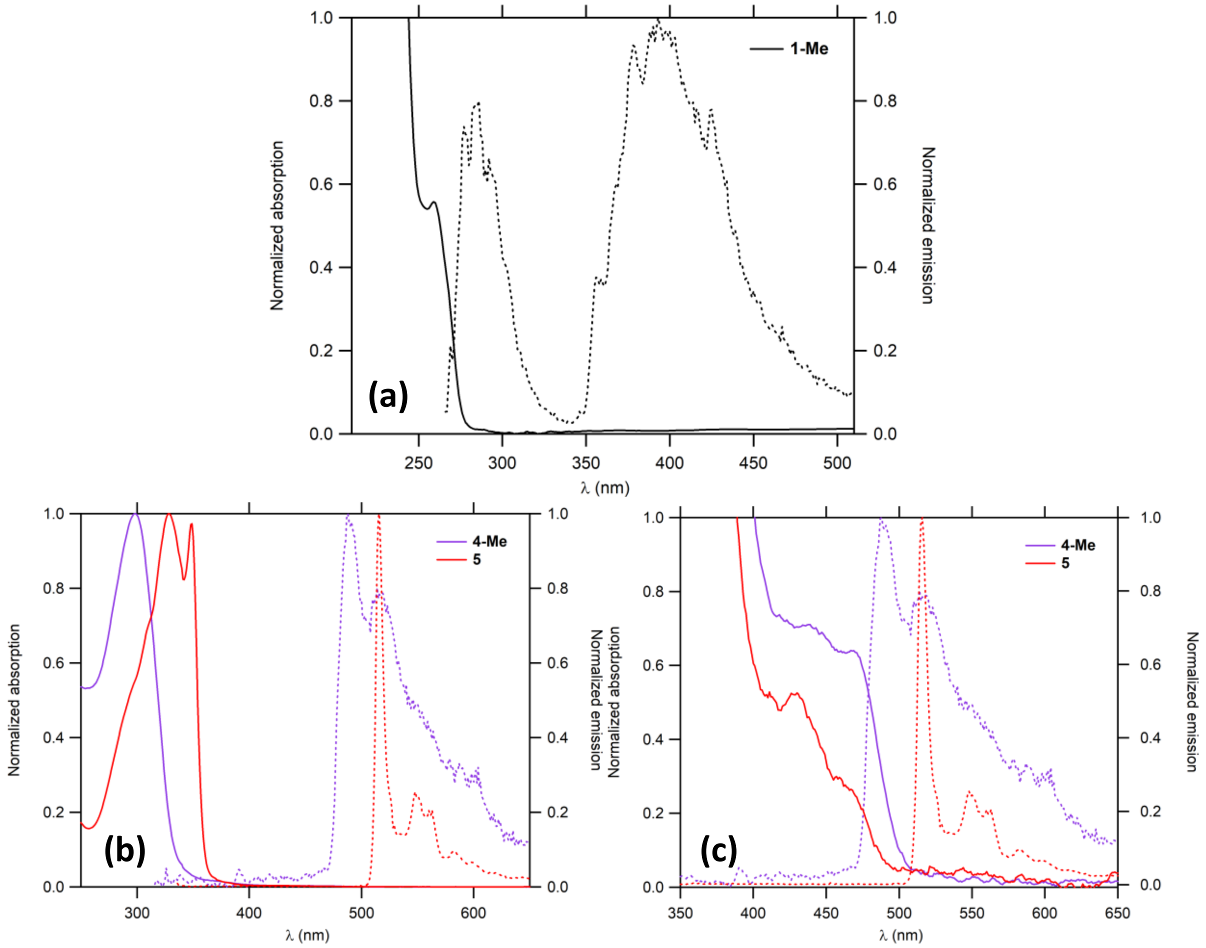

Figure S13. Absorption and emission spectra of compounds **1-Me** (a), **4-Me** and **5** (b) in EtOH at 77K. (c) *Ibid* with increased scale for absorption spectra of **4-Me** and **5**.

6. Cartesian coordinates of the DFT optimized geometries for 1-Me, 4-Me, 5', 6-Me and 7-Me

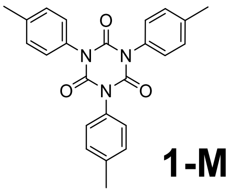

1-Me

C	-3.47463500	-4.36804000	0.00209100
C	-3.02986500	-3.80715600	-1.19914100
H	-3.36282900	-4.23056300	-2.14329500
C	-2.16677900	-2.71759000	-1.20614700
H	-1.82190800	-2.28601000	-2.14102600
C	-3.02473100	-3.81013000	1.20276900
H	-3.35365500	-4.23587400	2.14728600
C	-2.16166200	-2.72054400	1.20881800
H	-1.81291900	-2.29127700	2.14333500
C	-1.73847700	-2.18064400	0.00109000
N	-0.84301200	-1.05717600	0.00051100
C	0.52038900	-1.32533200	-0.00003000
O	0.96284800	-2.45231400	-0.00067000
C	5.51841800	-0.83925100	-0.00153200
C	4.80901600	-0.73818000	-1.20230000
H	5.33959700	-0.82856100	-2.14667700
C	3.43528900	-0.52615300	-1.20855300
H	2.88748100	-0.45012000	-2.14305900
C	4.81115800	-0.72807000	1.19961100
H	5.34341600	-0.81050300	2.14377000
C	3.43741700	-0.51612200	1.20649600
H	2.89119800	-0.43233300	2.14126800
C	2.75812500	-0.41566600	-0.00087200
N	1.33749500	-0.20160900	-0.00038200
C	0.88817600	1.11309900	-0.00068500
O	1.64335300	2.05944400	-0.00196200
C	-2.03336800	5.19821500	-0.00023600
C	-1.78230700	4.52724300	-1.20112400
H	-1.98236700	5.02701100	-2.14543900
C	-1.27857000	3.23177400	-1.20751900
H	-1.08296100	2.71492500	-2.14232200
C	-1.75891700	4.53650700	1.20064400
H	-1.94057800	5.04354400	2.14479900
C	-1.25518900	3.24100300	1.20729400
H	-1.04156400	2.73142100	2.14214700
C	-1.01907100	2.59613400	-0.00005400
N	-0.49373500	1.25888300	-0.00011600
C	-1.40787900	0.21231000	0.00050800
O	-2.60508400	0.39296700	0.00103100
C	7.00746200	-1.03745000	-0.00202900
H	7.33625900	-1.59011800	-0.88719700
H	7.52849600	-0.07192800	-0.00667400
H	7.33786300	-1.58244400	0.88728900
C	-2.60714100	6.58652400	-0.00008900
H	-2.30406200	7.14382800	-0.89152400
H	-3.70377800	6.55419700	0.00854700
H	-2.29015500	7.14943900	0.88292800
C	-4.43189200	-5.52572700	0.00267400
H	-4.29962100	-6.15271200	0.88949800
H	-5.47040300	-5.17185400	0.00387300
H	-4.30148900	-6.15204500	-0.88490800

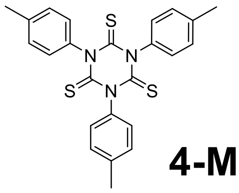

4-Me

C	-4.26535700	-3.59297000	-0.00001700
C	-3.71622800	-3.13371700	-1.20164600
H	-4.12459700	-3.48465400	-2.14578200
C	-2.65532800	-2.23711600	-1.20978400
H	-2.22879100	-1.88292700	-2.14318600
C	-3.71640700	-3.13341800	1.20161400
H	-4.12492700	-3.48413000	2.14577000
C	-2.65553200	-2.23683000	1.20969400
H	-2.22913800	-1.88240400	2.14307200
C	-2.13662300	-1.79763900	-0.00006800
N	-1.03172700	-0.86893700	-0.00008800
C	0.24759600	-1.39479300	0.00009200
C	5.24192000	-1.90493900	0.00019000
C	4.56812100	-1.66308000	-1.20148900
H	5.07237300	-1.85196800	-2.14562100
C	3.26327200	-1.18701600	-1.20963900
H	2.74105600	-1.00162400	-2.14320200
C	4.56825400	-1.66246400	1.20172500
H	5.07258000	-1.85084000	2.14591100
C	3.26333300	-1.18637000	1.20974100
H	2.74122100	-1.00049200	2.14326400
C	2.62489500	-0.95264600	0.00004400
N	1.26793800	-0.46092000	-0.00004200
C	1.08387800	0.90987500	-0.00029300
C	-0.96904300	5.49061200	0.00028900
C	-0.84263100	4.78601800	-1.20140100
H	-0.93109400	5.31711500	-2.14556500
C	-0.60368900	3.41772200	-1.20963100
H	-0.50384600	2.87271300	-2.14321900
C	-0.84239200	4.78585000	1.20179400
H	-0.93068600	5.31678600	2.14605900
C	-0.60344300	3.41750400	1.20975800
H	-0.50341700	2.87235900	2.14324700
C	-0.48732000	2.74766000	0.00001600
N	-0.23499800	1.32656600	-0.00009800
C	-1.33005200	0.48163100	-0.00015600
C	6.66355700	-2.38944700	0.00000700
H	6.87755800	-2.99897400	-0.88306100
H	7.36285600	-1.54401600	-0.00889400
H	6.88265500	-2.98491300	0.89132100
C	-1.25893800	6.96431700	0.00029800
H	-0.84099700	7.45355800	-0.88462300
H	-2.34061000	7.14818800	-0.00450300
H	-0.84896000	7.45201400	0.88975300
C	-5.43294000	-4.53777200	0.00010600
H	-5.42697300	-5.17988600	0.88597800
H	-6.38079500	-3.98513200	0.00305600
H	-5.43020600	-5.17617900	-0.88843600
S	-2.88179100	1.04500800	-0.00031200
S	2.34749500	1.97203800	-0.00063900
S	0.53634800	-3.02000700	0.00049900

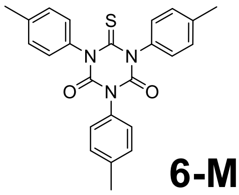

6-Me

C	-4.98481300	-2.42600700	0.00552100
C	-4.33077600	-2.13131900	1.20611200
H	-4.81233300	-2.37036200	2.15079100
C	-3.07604900	-1.53441900	1.21214800
H	-2.57070200	-1.30674200	2.14582400
C	-4.35039300	-2.09522900	-1.19597700
H	-4.84723800	-2.30585600	-2.13948800
C	-3.09566100	-1.49799100	-1.20438200
H	-2.60566100	-1.24215800	-2.13895900
C	-2.46491600	-1.23049700	0.00304100
N	-1.17683800	-0.58656300	0.00177600
C	-1.20901900	0.81147100	0.00839700
O	-2.24600400	1.43298600	0.01727800
C	0.12886700	5.65228500	0.00106700
C	0.14908000	4.93612300	1.20205700
H	0.18733600	5.47417500	2.14568000
C	0.11869800	3.54659100	1.20981100
H	0.13211000	2.99376700	2.14430300
C	0.07073000	4.93688400	-1.19898600
H	0.04740500	5.47538600	-2.14283600
C	0.04100600	3.54722700	-1.20542700
H	-0.00497100	2.99480800	-2.13913600
C	0.06581300	2.86224900	0.00240100
N	0.03248300	1.42518600	0.00244200
C	1.24441100	0.75490400	-0.00401600
O	2.30913600	1.32767700	-0.00848700
C	4.86274900	-2.66047100	-0.00144900
C	4.23198300	-2.31899900	1.19907200
H	4.70735900	-2.56962400	2.14388200
C	3.00808300	-1.66099100	1.20485700
H	2.52048500	-1.39740700	2.13853600
C	4.23738300	-2.31385300	-1.20336800
H	4.71708500	-2.56050600	-2.14703200
C	3.01350400	-1.65579800	-1.21206600
H	2.53019400	-1.38828300	-2.14682900
C	2.40452500	-1.34279900	-0.00430400
N	1.14775300	-0.63993700	-0.00447000
C	-0.03149800	-1.35173100	-0.00578100
C	0.19319200	7.15295000	-0.00020500
H	-0.27332600	7.57331300	0.89577000
H	1.23473100	7.49727200	-0.01894700
H	-0.30574400	7.57326000	-0.87846000
C	6.16962500	-3.40090900	0.00004200
H	6.76140700	-3.16475800	0.88951300
H	6.00233300	-4.48525400	-0.00320200
H	6.76613900	-3.16016200	-0.88502100
C	-6.32660000	-3.10111900	0.00661800
H	-6.91041600	-2.83090500	-0.87836900
H	-6.21296100	-4.19238500	0.00322400
H	-6.90615600	-2.83644500	0.89615700
S	-0.06909100	-3.00741000	-0.01594100

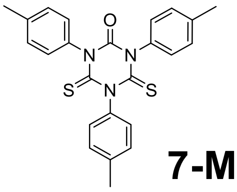

7-Me

C	-5.50567600	-0.15054100	-0.00008300
C	-4.78991300	-0.13294800	-1.20156300
H	-5.32796400	-0.15293900	-2.14574500
C	-3.40141400	-0.09255200	-1.20931500
H	-2.84700200	-0.08116600	-2.14266500
C	-4.78998500	-0.13251400	1.20146300
H	-5.32809900	-0.15218000	2.14561800
C	-3.40150900	-0.09211700	1.20928700
H	-2.84714700	-0.08039600	2.14266400
C	-2.72120300	-0.07185500	-0.00000300
N	-1.27854800	-0.03476900	0.00008000
C	-0.60655900	-1.24728800	0.00004200
C	3.14141900	-4.65182000	0.00011400
C	2.73797700	-4.06034200	-1.20127900
H	3.04023700	-4.50576600	-2.14553700
C	1.95544900	-2.91214800	-1.20890200
H	1.63976800	-2.45748100	-2.14296900
C	2.73713700	-4.06096100	1.20144000
H	3.03871400	-4.50684300	2.14569200
C	1.95458400	-2.91269200	1.20908400
H	1.63826600	-2.45850300	2.14316600
C	1.56465900	-2.35302500	0.00011500
N	0.76581400	-1.15466400	0.00007600
C	1.48135600	0.03971700	0.00006700
O	2.68894600	0.07196100	-0.00005100
C	2.90613100	4.80238100	0.00018400
C	2.53404700	4.19064700	-1.20118400
H	2.82390300	4.64433800	-2.14536300
C	1.80271900	3.00916600	-1.20887100
H	1.51596200	2.53581300	-2.14298600
C	2.53328800	4.19113700	1.20151900
H	2.82252600	4.64520100	2.14570500
C	1.80194000	3.00962100	1.20920400
H	1.51460400	2.53663600	2.14332700
C	1.43611400	2.43371400	0.00017700
N	0.70266400	1.19404400	0.00020000
C	-0.67264700	1.21200600	-0.00005800
C	4.01162000	-5.87609400	-0.00014800
H	3.82878200	-6.49565600	-0.88322400
H	5.07319300	-5.59900000	-0.00901300
H	3.84110000	-6.48737900	0.89105900
C	3.66948000	6.09595200	0.00003500
H	4.30672100	6.18352400	-0.88506700
H	2.98347700	6.95218900	-0.00472200
H	4.29996200	6.18776200	0.88950700
C	-7.00764200	-0.16265000	-0.00003500
H	-7.40284900	-0.66923300	0.88561500
H	-7.40526600	0.85998600	0.00332900
H	-7.40297500	-0.66358200	-0.88881400
S	-1.53710200	2.62071500	-0.00066300
S	-1.39260000	-2.70146900	0.00007500

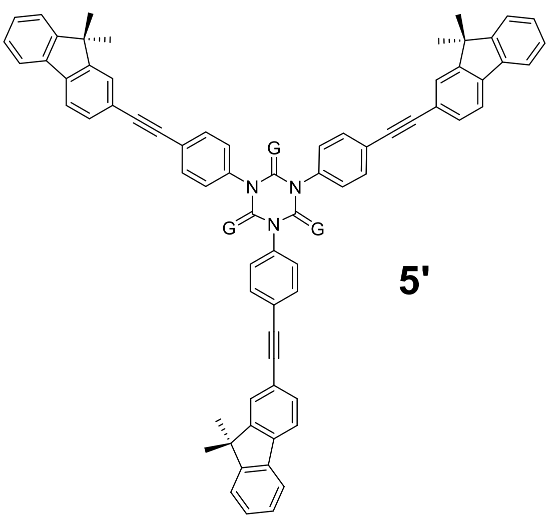

5'

Conformation C1:

C	-5.26389400	-6.23428500	0.62936100
C	-4.47601900	-5.31468900	0.51606500
C	-3.55834700	-4.23956600	0.38368000
C	-3.02888800	-3.60942200	1.52369900
H	-3.32562800	-3.95239000	2.50925600
C	-2.13649900	-2.55757800	1.39209900
H	-1.72437400	-2.06490800	2.26686200
C	-3.17031700	-3.79122600	-0.89125700
H	-3.57634100	-4.27493300	-1.77340300
C	-2.27762200	-2.73998500	-1.02236200
H	-1.97356300	-2.38726300	-2.00258200
C	-1.77102300	-2.13376400	0.12048600
N	-0.84557800	-1.03643400	-0.01849500
C	-1.37411000	0.23998300	-0.08630500
C	-2.81374800	7.67183000	-0.59064400
C	-2.39043900	6.53358900	-0.52392600
C	-1.89636800	5.20467500	-0.44988300
C	-1.57699100	4.63376900	0.79478400
H	-1.70935900	5.21780100	1.69943300
C	-1.09787400	3.33581800	0.86797300
H	-0.84992600	2.88730100	1.82458700
C	-1.72285100	4.44345600	-1.61927500
H	-1.96816300	4.88017500	-2.58162100
C	-1.24407600	3.14533500	-1.54558000
H	-1.10847700	2.55055800	-2.44316200
C	-0.93703200	2.60543900	-0.30280600
N	-0.44261900	1.25284900	-0.22605300
C	0.92596500	1.06647900	-0.29896000
C	8.07332300	-1.48340600	-0.58881100
C	6.87906300	-1.26307900	-0.52243800
C	5.48481700	-1.00630100	-0.44709800
C	4.86971200	-0.77967100	0.79680100
H	5.46951700	-0.80159200	1.70054900
C	3.50872200	-0.53095200	0.87050700
H	3.02619500	-0.35538500	1.82655900
C	4.70285800	-0.97623900	-1.61536100
H	5.17361100	-1.15013200	-2.57721500
C	3.34190100	-0.72711900	-1.54123000
H	2.73126300	-0.70260300	-2.43811200
C	2.75938000	-0.50746500	-0.29912600
N	1.34228300	-0.25047200	-0.22279000
C	0.50226500	-1.34025200	-0.08096900
C	9.46771100	-1.74122300	-0.65943200
C	10.24295100	-1.76577400	0.51703200
C	10.08104700	-1.97401800	-1.90579100
C	11.59786200	-2.01901100	0.42669200
H	9.76282500	-1.58556800	1.47501900
C	11.44300800	-2.22838500	-1.99194900
H	9.47086800	-1.95193500	-2.80337100
C	12.20201400	-2.25067000	-0.82260900
H	11.90257900	-2.40652800	-2.96029100
C	-6.18348800	-7.30863600	0.75580800
C	-6.71256300	-7.92477400	-0.39583500
C	-6.57164100	-7.76141300	2.03173400
C	-7.60743200	-8.96725300	-0.25241700
H	-6.40864500	-7.56963700	-1.37676400
C	-7.47095900	-8.80968300	2.17128300
H	-6.15586700	-7.27686400	2.90968800
C	-7.98934200	-9.41304300	1.02620800
H	-7.76151600	-9.14845000	3.16195800
C	-8.93554700	-10.51542000	0.86741000
C	-9.60015800	-11.29088700	1.81577200
C	-9.12812800	-10.73831000	-0.50707400
C	-10.46083800	-12.29317700	1.37419200
H	-9.45278600	-11.12058700	2.87911800
C	-9.98638900	-11.73795600	-0.94060400
C	-10.65298600	-12.51560700	0.00865100
H	-10.98790800	-12.90807500	2.09839900
H	-10.14204400	-11.91833200	-2.00167200
C	-8.30049800	-9.77297500	-1.33727700
C	-3.30658300	9.00138200	-0.66300600
C	-3.51117900	9.74485700	0.51647500
C	-3.59434400	9.58149400	-1.91373100
C	-3.99324800	11.03612400	0.42472600
H	-3.28719900	9.29061000	1.47783800
C	-4.07810800	10.87970300	-2.00136000
H	-3.43190600	8.99640500	-2.81358700
C	-4.27776900	11.60757600	-0.82905600
H	-4.29568900	11.31423900	-2.97319900
C	-4.76766000	12.96789100	-0.61609700
C	-5.18006700	13.94156000	-1.52402200
C	-4.78078600	13.22178600	0.76642500
C	-5.60624000	15.17382900	-1.03389400
H	-5.17088300	13.74766600	-2.59346900
C	-5.20621700	14.45087000	1.24839600
C	-5.61958000	15.42733800	0.33966100
H	-5.93141200	15.94545000	-1.72616600
H	-5.21990000	14.65715500	2.31605400
C	-4.29016300	12.01513100	1.54709600
C	13.62814100	-2.48776900	-0.60898200
C	14.65270100	-2.76193800	-1.51314600
C	13.88935600	-2.40025200	0.76947400
C	15.94332200	-2.94823300	-1.02332100
H	14.45329700	-2.82993000	-2.57945700
C	15.17661400	-2.58690400	1.25115600
C	16.20407300	-2.86185100	0.34622200
H	16.75517000	-3.16290800	-1.71266500
H	15.38900600	-2.52164300	2.31569300
C	12.62107900	-2.09371200	1.54639800
H	17.21681700	-3.01019700	0.71068900
H	-5.95505400	16.39429600	0.70427200
H	-11.32809700	-13.30182400	-0.31781400
C	12.28498500	-3.21965800	2.53295400
H	11.33548700	-3.01510500	3.03977300

H	12.20125600	-4.18121500	2.01698400
H	13.06581600	-3.30389600	3.29668900
C	12.73180900	-0.75517900	2.28827000
H	12.96893100	0.05851500	1.59580300
H	11.78892700	-0.51659800	2.79255000
H	13.51988900	-0.80356500	3.04766400
C	-7.28398500	-10.51950300	-2.21112800
H	-6.65360800	-9.80993800	-2.75829000
H	-6.63701400	-11.15805700	-1.60161600
H	-7.80026300	-11.14947800	-2.94372300
C	-9.19328000	-8.88004100	-2.20893900
H	-9.92251500	-8.33893800	-1.59799100
H	-8.58798200	-8.14790900	-2.75468000
H	-9.73769100	-9.48439000	-2.94273700
C	-5.38088500	11.47481200	2.48133700
H	-6.29061000	11.23247600	1.92320400
H	-5.03562400	10.56833900	2.99059400
H	-5.63191600	12.21852700	3.24548000
C	-3.02368700	12.34586400	2.34750300
H	-2.64644200	11.45178100	2.85596300
H	-2.23511000	12.72993300	1.69293100
H	-3.24024900	13.10310200	3.10895500
S	1.06596500	-2.88764900	0.00664000
S	-2.99511100	0.53289200	-0.00743400
S	1.98319000	2.32168400	-0.46179200

Conformation C2:

C	1.26199244	8.09981960	0.55301193
C	1.07281422	6.89839076	-0.55008512
C	0.85381144	5.49564755	-0.54882851
C	0.74292124	4.79399593	0.66465961
H	0.81535583	5.33333640	1.60232094
C	0.53028437	3.42486234	0.66546093
H	0.44329559	2.87592421	1.59763728
C	0.74654842	4.79178504	-1.76133244
H	0.83177140	5.32950103	-2.69973340
C	0.53374064	3.42270929	-1.76021121
H	0.44950798	2.87215649	-2.69167637
C	0.42819326	2.75398619	-0.54693010
N	0.20628804	1.32879189	-0.54640213
C	6.40757103	-5.12292265	-0.56185009
C	5.44951017	-4.36120163	-0.55713175
C	4.34336534	-3.48134560	-0.55419322
C	3.77956231	-3.04866643	0.65997920
H	4.20465582	-3.40232193	1.59714291
C	2.69997951	-2.18015969	0.66216908
H	2.25688594	-1.84312846	1.59471175
C	3.78856369	-3.02284919	-1.76582550
H	4.22275939	-3.35197645	-2.70488724
C	2.70903814	-2.16454298	-1.76314736
H	2.28512601	-1.80306203	-2.69380093
C	2.17150598	-1.75260712	-0.54937473
N	1.04825710	-0.84774919	-0.54739304
C	-7.65089854	-2.95628085	-0.56990197
C	-6.51546202	-2.52044351	-0.56390245
C	-5.19078430	-2.00968167	-0.55964203
C	-4.52993089	-1.76530216	0.65516051
H	-5.03026397	-1.96316032	1.59185209
C	-3.23760712	-1.26563164	0.65860831
H	-2.71070439	-1.06615634	1.59183525
C	-4.52465272	-1.75150481	-1.77073151
H	-5.03083451	-1.94661385	-2.71025433
C	-3.23239956	-1.25181554	-1.76685376
H	-2.71137774	-1.04955598	-2.69716119
C	-2.60314495	-1.00863942	-0.55234170
N	-1.25784647	-0.48828826	-0.54898121
C	-8.97654110	-3.46476593	-0.56267064
C	-9.63695044	-3.72741917	0.65413895
C	-9.63656150	-3.70800035	-1.78291109
C	-10.92692804	-4.22095908	0.62846037
H	-9.12140041	-3.53759923	1.59167427
C	-10.93286695	-4.20407239	-1.80407842
H	-9.11497196	-3.50087574	-2.71216238
C	-11.57850166	-4.46069011	-0.59526826
H	-11.42954176	-4.38663948	-2.75306800
C	1.48290402	9.50235087	-0.56118703
C	1.58754562	10.20099761	0.65811847
C	1.59879592	10.20046008	-1.77897464
C	1.80357306	11.56523974	0.63722459
H	1.49681123	9.65604612	1.59383598
C	1.81608833	11.57141069	-1.79527959
H	1.51581564	9.64872014	-2.71019001
C	1.91882438	12.25423561	-0.58402415
H	1.90348123	12.09665890	-2.74242298
C	2.14273563	13.66951027	-0.29645141
C	2.32095235	14.75677202	-1.15001130
C	2.16210973	13.83952502	1.09882461
C	2.51875798	16.01788290	-0.59250051
H	2.30683875	14.62794276	-2.22919450
C	2.35947277	15.09783068	1.64796174
C	2.53790828	16.18810562	0.79377554
H	2.65954463	16.87758230	-1.24171772
H	2.37627960	15.23973040	2.72598253
C	1.94789298	12.51630032	1.81240839
C	0.67277391	12.54294937	2.66546591
H	-0.19969172	12.80369733	2.05830318
H	0.49575994	11.57291196	3.12207197
H	0.76669750	13.29030090	3.47018737
C	3.15850621	12.14834798	2.68034890
H	3.01693682	11.16366152	3.13919066
H	4.07554399	12.12284418	2.08349323
H	3.28931438	12.88135954	3.48388773
C	7.52496779	-6.00123270	-0.56223647
C	8.01828651	-6.51527882	0.65580497
C	8.13426253	-6.36239215	-1.78104432
C	9.10498607	-7.37021557	0.63258732
H	7.53941052	-6.23065295	1.59230412
C	9.22620354	-7.22162905	-1.79967211
H	7.74758594	-5.96149631	-2.71124803
C	9.70665949	-7.72624110	-0.58976162
H	9.68955341	-7.49338501	-2.74761069
C	10.82325619	-8.62502786	-0.30481208
C	11.72881166	-9.25546074	-1.16023798
C	10.88975980	-8.82404571	1.08997071
C	12.70380523	-10.07724809	-0.60509950
H	11.68506812	-9.10319740	-2.23905349
C	11.86251204	-9.64403713	1.63678918
C	12.77036192	-10.26629459	0.78073485
H	13.43024907	-10.57313940	-1.25578457
H	11.92592558	-9.79515142	2.71447414
C	9.79783504	-8.04406242	1.80585771
C	8.82708334	-8.99557883	2.54914455
H	8.01301844	-8.42547057	3.00964450

H	8.39942743	-9.73565512	1.85881430
H	9.35798571	-9.52699222	3.35119003
C	10.38476653	-7.01560791	2.78400837
H	11.07398217	-6.32491206	2.27009100
H	9.58246201	-6.41785421	3.25346112
H	10.93834176	-7.51786084	3.58657845
C	-12.91686702	-4.97529526	-0.31290540
C	-13.94506667	-5.36248137	-1.17044091
C	-13.07768547	-5.04745616	1.08171470
C	-15.13760205	-5.82349371	-0.61758147
H	-13.82325144	-5.30772544	-2.24913669
C	-14.26763566	-5.50726156	1.62622791
C	-15.29858243	-5.89562596	0.76804850
H	-15.95058388	-6.12990786	-1.26992518
H	-14.40176622	-5.56715644	2.70371640
C	-11.82620006	-4.57440797	1.80013496
C	-12.10422799	-3.34411230	2.67075228
H	-11.18153083	-2.97629815	3.13293778
H	-12.53861716	-2.53522068	2.07506670
H	-12.80659310	-3.59906161	3.47185164
C	-11.21530943	-5.69493697	2.65188309
H	-11.00315301	-6.57917845	2.04281548
H	-10.27971358	-5.35998725	3.11300248
H	-11.90399092	-5.98427400	3.45320530
H	-16.23563669	-6.25779709	1.19191734
H	13.54216048	-10.91629804	1.19641464
H	2.69354474	17.17904643	1.21126498
C	-1.10406135	0.88618163	-0.54755056
C	-0.21903703	-1.40144891	-0.55916027
C	1.31974400	0.50825272	-0.54613535
S	-2.38840907	1.92085686	-0.54650715
S	-0.47281487	-3.03088803	-0.56149777
S	2.85806115	1.10290685	-0.53457119

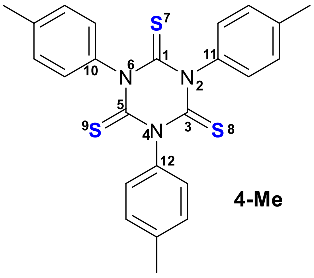

4-Me

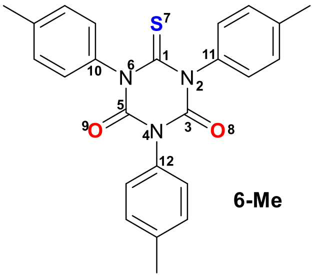

6-Me

Table S4. Comparison of selected experimental and theoretical structural parameters for compounds **4-Me** and **6-Me** (see molecular scheme above).

**Bonding parameters**		**4-Me** Exp.	**4-Me** Opt.	**Bonding parameters**	**6-Me** Exp.	**6-Me** Opt.
**Bond lengths (Å)**	**C1-N2**	1.38	1.38	**C1-N2**	1.39	1.37
	**N2-C3**	1.39	1.38	**N2-C3**	1.39	1.40
	**C3-N4**	1.39	1.38	**C3-N4**	1.39	1.38
	**N4-C5**	1.39	1.38	**N4-C5**	1.39	1.38
	**C5-N6**	1.39	1.38	**C5-N6**	1.39	1.40
	**N6-C1**	1.39	1.38	**N6-C1**	1.39	1.37
	**C1-S7**	1.64	1.65	**C1-S7**	1.67	1.65
	**C3-S8**	1.65	1.65	**C3-O8**	1.20	1.21
	**C5-S9**	1.64	1.65	**C5-O9**	1.25	1.21
	**N2-C11**	1.46	1.44	**N2-C11**	1.46	1.44
	**N4-C12**	1.46	1.44	**N4-C12**	1.45	1.44
	**N6-C10**	1.46	1.44	**N6-C10**	1.46	1.44
**Bond angles (°)**	**S7-C1-N2**	122.71	122.40	**S7-C1-N2**	126.10	122.41
	**S8-C3-N4**	122.51	122.41	**O8-C3-N4**	123.70	122.77
	**C12-N4-C5**	117.34	117.59	**C12-N4-C5**	116.84	117.63
	**S9-C5-N6**	122.85	122.39	**O9-C5-N6**	122.58	122.25
**Torsion angles (°)**	**S7-C1-N2-C11**	-8.33	0.00	**S7-C1-N2-C11**	11.57	-0.35
	**S8-C3-N4-C12**	-3.08	-0.00	**O8-C3-N4-C12**	-8.22	0.06
	**C12-N4-C5-O9**	-0.97	-0.22	**C12-N4-C5-O9**	8.01	0.21
	**S9-C5-N6-C10**	-0.32	0.00	**O9-C5-N6-C10**	-0.59	0.14

7. Selected frontier MOs for 1-Me, 4-Me, 6-Me, 7-Me and 5’

**1-Me**	
**LUMO+4**<br>(Ph)	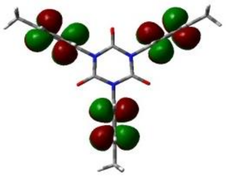
**HOMO-4**<br>(Ph)	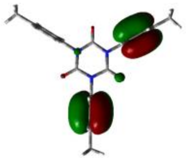
**HOMO-5**<br>(Ph)	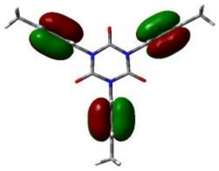
**4-Me**	
**HOMO-4**<br>(Ph/Tiso)	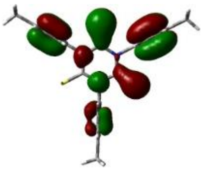
**HOMO-5**<br>(Ph/Tiso)	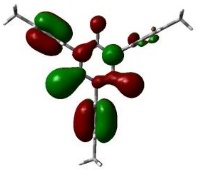
**5’**	
**LUMO+4**<br>(Flu+Ph)	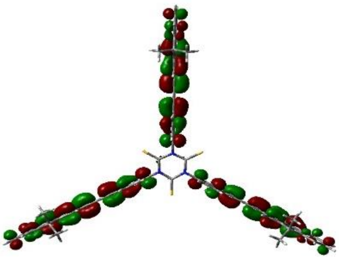
**LUMO+3**<br>(Flu+Ph)	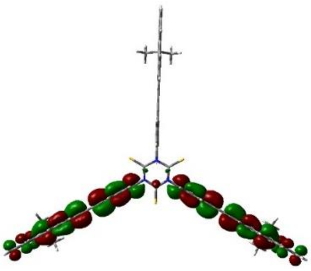

6-Me	
**HOMO-6**<br>(PH/TISO)	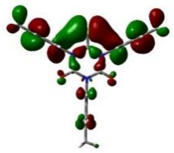
**HOMO-7**<br>(PH/TISO)	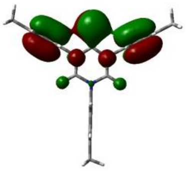
**7-Me**	
**HOMO-4**<br>(PH/TISO)	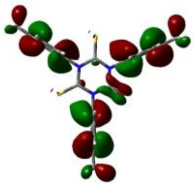

Figure S14. Frontier MOs of **1-Me**, **4-Me**, **5**, **6-Me** and **7-Me**. Contour values are ± 0.03 (e/bohr^3)$^{1/2}$.

8. Computed atomic charges and bond orders for 1-Me and 4-Me

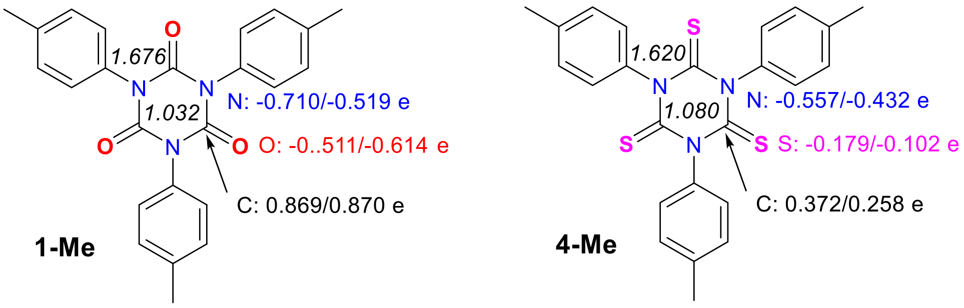

Figure S15. Computed atomic charges (Mulliken/NBO) and bond orders (Wiberg; in italics) for **1-Me** and **4-Me**.

9. Computed dipole moments for 1-Me, 4-Me, 5', 6-Me and 7-Me

Table S5. Calculated dipole moment for compounds **1-Me**, **3'**, **4-Me**, **5'**, **6-Me** and **7-Me**.

Cpds	μ (D)			
	μ_x	μ_y	μ_z	$\lvert\mu\rvert$
**1-Me**	0.080	0.057	0.002	0.099
**3'** [C1] [a,b]	0.030	0.010	0.770	0.77
**3'** [C2] [a,c]	-0.000	0.009	2.316	2.32
**4-Me**	-0.062	0.065	-0.001	0.090
**5'** [C1] [b]	-0.010	0.060	0.720	0.73
**5'** [C2] [c]	0.030	-0.083	2.311	2.31
**6-Me**	0.087	1.617	0.021	1.620
**7-Me**	1.394	0.145	-0.00	1.402

[a] In **3'** the butyl chains of **3** have been replaced by methyl groups to expedite the DFT calculations. [b] Statistically favoured conformation C1 with only two arms pointing on the same side of the central ring. [c] Conformation C2 with all arms pointing on the same side of the central ring.

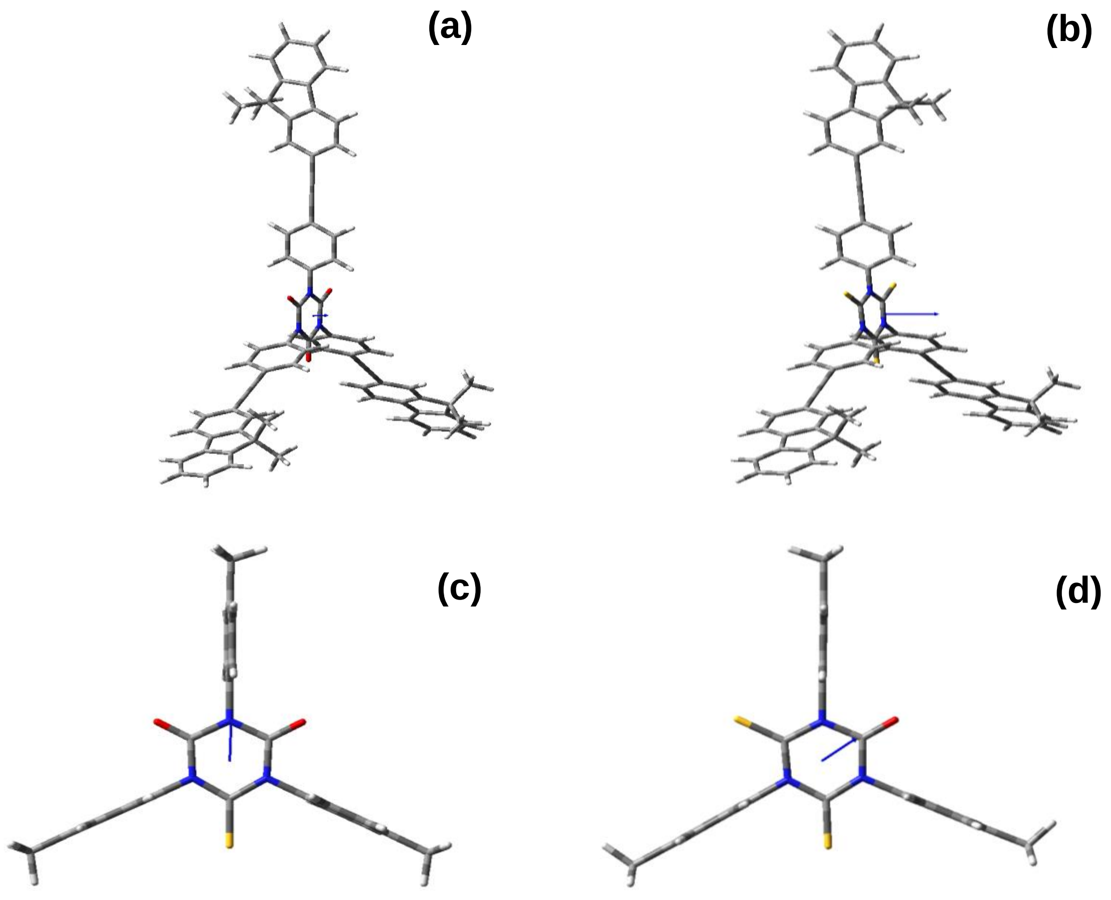

Figure S16. Dipole moments of **5'** in conformation C1 (a) and C2 (b)**, 6-Me** (c) and **7-Me** (d).

10. Computed vibrational spectra for 1-Me, 4-Me, 6-Me and 7-Me

Table S6. Most intense experimentally observed *vs.* calculated IR- and Raman-active vibrational modes for **1-Me**, **4-Me**, **6-Me** and **7-Me**. The vibrational modes involving the largest contributions from the ν(C=X) vibrators (X = O, S) are marked in bold characters for each compound.

Vibrational Modes				
Nature of major vibrational motions[a]	Rescaled computed frequencies[b,c]	Computed intensites: IR-active modes[d]	Computed intensites: Raman-active modes[e]	Exp. Frequencies[b] IR/Raman
		**1-Me**		
**ν(C=O)$_s$**	1806	0	185	1773 (vw)/1772 (s)
**ν(C=O)$_{as}$**	1739 (deg)	1050, 1049	<5	[1716 (s),1694 (vs)][f]/no
ν(C=C)$_{Ar}$	1628 (deg)	0, 11, 10	230, 100, 112	no/1610-1590 (m)
ρ(C$_{Ar}$-H)	1512 (deg)	143, 146	5, 4, 6	1511 (m)/1513 (vw)
δ(CH$_3$)	1446 (deg)	9, 11, 8	34, 32, 36	no/1438 (w)
ν(C-N)	1422 (deg)	1184, 1177	58, 60	1401 (vs)/1408 (w)
ω(CH$_3$)	1376 (deg)	<1	68, 65, 73	no/1376 (m)
ν(C-N)	1315	0	115	no/1306 (m)
ν(C-N)	1246 (deg)	37, 35	44, 45	1229 (w)/1232 (w)
ρ(C$_{Ar}$-H)	1207	9, 10	65	1208 (vw)/1210 (s)
ν(C-N)	1046	0	63	no/1009 (s)
ν(C-N)	1008	<1	37	no/1031 (m)
Ar-breathing	855 (deg)	9, 5	13, 12	827 (vw)/828 (vw)
δ(C$_{Ar}$-H)	809	4	74	no/798 (vs)
δ(C$_{Ar}$-H) (OPCO)	801(deg)	47, 34	8, 8	806 (m)/no
δ(C-N) (OPCO)	754 (deg)	79-83	0; 0	748 (s)/no
δ(C-N) + **ν(C=O)$_s$** (SCBM)	679	0	56	no/676 (s)
Ar-deformation	634	0	77-77	no/642 (m)
Ar-oscillation	536 (deg)	77-77	4, 3	or/no
		**4-Me**		
ν(C=C)$_{Ar}$	1625 (deg)	3, 2, 1	231, 283, 365	no/1604 (m)
ρ(C$_{Ar}$-H)	1512	1	55	no/no
ρ(C$_{Ar}$-H)	1507 (deg)	122-121	15	1508 (m)/no
ω(CH$_3$)	1376 (deg)	2, 4, 1	96, 115	no/1375 (m)
ν(C-N)	1372 (deg)	2210, 2213	466, 469	1351 (vs)/1354 (vw)
ρ(C-N) + ν(C=S)$_s$	1326	0	363	no/1329 (vs)
ν(C-N) + **ν(C=S)$_{as}$**	1271 (deg)	918, 922	11, 10	1251(s)/no)
ν(C-N)+**ν(C=S)$_s$**	1230	<1	30	no/1266 (w)
ρ(C$_{Ar}$-H) + ν(C=S)$_s$	1204	0	106	no/1203 (m)
ρ(C$_{Ar}$-H) + ν(C=S)$_{as}$	1182	56, 58	6, 5	1182 (w)/no
δ(C$_{Ar}$-H)	1050	34, 11, 18	2, 2, 2	1020 (w)/no
ρ(C$_{Ar}$-H) + ν(C-N)	1044	<1	113	no/1030 (m)
ρ(C-N)	1006	<1	30	no/1004 (w)
δ(C$_{Ar}$-H)	953 (deg)	1, 19, 20	0, 2, 2	939 (w)/no

ρ(C-N) + ν(C=S)$_{as}$	943 (deg)	15, 10	20, 21	no/931 (vw)
δ(C$_{Ar}$-H)	832	1	30	no/no
δ(C$_{Ar}$-H)	805 (deg)	20, 20, 19	37, 37, 45	797 (m)/797 (s)
ρ(C-N) + δ(C$_{Ar}$-H) (IPCO)	728, 732, 740	90, 90, 14	18, 21, 2	726 (m)/no
π(C=S)$_{as}$	611 (deg)	0, 0	23, 23	or/no
δ(C-N) + **ν(C=S)$_s$** (SCBM)	453	0	65	or/436 (s)
ρ(NC=S)	286 (deg)		22, 23	or/no
**6-Me**				
**ν(C=O)$_s$**	1788	312	210	1753 (w)/1755 (s)
**ν(C=O)$_{as}$**	1743	1179	1	[1730(m), 1708 (s)][e] /no
ν(C=C)$_{Ar}$	1627 (deg)	1, 6, 9	285, 133, 148	no/1605-1590 (m)
ρ(C$_{Ar}$-H)	1510 (deg)	134-130	2	1510 (m)/1509 (vw)
δ(CH$_3$)	1449 (deg)	12, 8, 23	33, 24, 30	no/1452 (w)
δ(CH$_3$) + δ(C$_{Ar}$-H)	1446 (deg)	9, 9, 8	31, 35, 37	no/no
ν(C-N)	1413	812	86	1411 (m)/no
ν(C-N)	1404	1831	159	1381 (vs) /1381 (vw)
ω(CH$_3$)	1376 (deg)	<1	75, 50, 97	no/no
ρ(C-N) + ν(C=S)	1317	285	126	1315 (m)/1315 (s)
ρ(C-N) + **ν(C=S)**	1297	485	20	1270 (s)/no
ν(C-N)	1241	22	43	no/1306
ρ(C-H)	1207 (deg)	4, 4, 6	36, 28, 33	no/1211 (s)
ρ(C-H) + ρ(C-N)	1052	16	65	no/1036 (s)
ρ(C-H)	1050 (deg)	23, 19, 16	1, 1, 2	
ν(C-N) + δ(C-H) + ν(C=S) (IPCO)	1029	29	3	1036 (vw)/no
ν(C-N) + δ(C-H) + ν(C=S) (IPCO)	1012	16	5	1021 (vw)/no
ν(C-N) + δ(C-H) + ν(C=S) (IPCO)	1001	22	1	no/986 (m)
ρ(C-N) + δ(C-N)	850	16	33	no/840 (vw)
δ(C$_{Ar}$-H)	814	46	10	803 (s)/no
ρ(C$_{Ar}$-H)	808	3	85	no/798 (vs)
δ(C-N) (OPCO)	749-751	100	1	744 (s)/no
δ(C-N) + **ν(C=O)$_s$** + **ν(C=S)** (SCBM)	608	<1	46	or /606 (m)
δ(C-H) (OPCO)	531	89	35	or/533 (w)
δ(C=S)	463	0	<1	or/479 (vw)
ρ(C=O) + ν(C=S) (IPCO)	414	16	12	or/416 (vw)
**7-Me**				
**ν(C=O)**	1767	79	186	1728 (s)/1728 (m)
ν(C=C)$_{Ar}$	1626 (deg)	1, 6, 6	253, 228, 209	no/1602-1590 (vs)
ρ(C$_{Ph}$-H)	1509 (deg)	131-127	5,15	1508 (m)/1512 (vw)
δ(CH$_3$)	1446 (deg)	8, 9, 7	35, 33, 37	1447 (vw)/1448 (w)
ν(C-N) + ν(C=S)$_s$	1404	1308	224	1403 (w)/1407 (vw)
ν(C-N)	1387	1874	255	1368 (vs)/no
ω(CH$_3$)	1375 (deg)	<1	76, 80, 94	no/1379 (m)
ν(C-N) + ν(C=S)$_s$	1318	28	224	no/1316 (s)
ν(C-N) + **ν(C=S)$_{as}$**	1295	1472	<1	1267 (s)/no
ν(C-N) + **ν(C=S)$_s$**	1261	130	50	1244 (s)/1246 (vw)

ρ(C-H) + ρ(C-N) + ν(C=S)$_s$	1187 (deg)	48, 34	38, 18	1210 (w)/1210 (m)
ρ(C-H)	1124	38	0	1108 (w)/no
ρ(C-N) + ν(NC=S)$_s$	1080	5	6	1071 (vw)/no
δ(CH_3)	1050 (deg)	20, 29, 15	2, 1, 2	1036-1060 (vw)/no
ν(C-N)	1042	1	87	no/1003 (w)
ν(C-N)	1007	4	37	no/1059 (s)
ρ(C-N) + δ(C-H) + ν(C=S)$_{as}$	944	22	6	941 (w)/931 (w)
δ(C_{Ar}-H)	832	4	32	825 (w)/815 (vw)
δ(C_{Ar}-H)	806/811/814	14, 27, 32	57, 46, 5	801 (s)/797 (s)
δ(C-N) + ν(C=O) + ν(C=S)$_s$ (SCBM)	748	32	<1	741 (m)/no
δ(C_{Ar}-H)	732	83	13	727 (s)/no
δ(C=S)$_s$	672	<5	<1	671 (vw) /no
δ(C=S)$_{as}$	609	0	22	or/612 (w)

[a] Vibrational motions; ν: stretching; ρ: in-plane bending (rocking); δ: out-of-plane bending (twisting); CH_3 umbrella inversion ; OPCO : Out-of-plane core oscillation; IPCO : In-plane core oscillation; SCBM: symmetric core breathing mode.[b] Frequencies in cm^{-1} (deg: degenerate band; or: out of range; no: not observed as an extremum). [c] Empirical scaling factors (0.96 for freq > 1200 cm^{-1}; 0.98 for 600 < freq < 1200 cm^{-1}; no scaling below 500 cm^{-1}). [d] IR activities in km/mol. [e] Raman activities in $Å^4$/AMU. [f] Fermi coupling.

11. Computed singlet lowest-lying transitions for 1-Me, 4-Me, 5', 6-Me and 7-Me

Table S7. Nature of the relevant first computed (PBE1PBE-GD3BJ /6-31G*) singlet excited states[a] (λ_{max} and λ_{cal} in nm, Oscillator Strength, excited state number (S_n), transition percentage and assignment) vs. experimental values (λ_{max}, ε_{max}) in CH_2Cl_2 (PCM).

Model Cpnd [Real Cpnd]	Experimental	Calculated for optimized geometry			Major Assignment [f]
	λ_{max} [ε_{max}] [b]	λ_{max} [c]	λ_{cal} [f] [d] (S_n) [e]	Composition	
**1-Me**	258 (sh) [0.7]	/	226 [0.00] (S_1)	H→L+2 (25%)<br>H-5→L (15%)<br>H-1→L+4 (13%)	$(\pi^*)_{Iso} \leftarrow (\pi)_{Ph+Iso}$
**[1-Me]**			226 [0.00] (S_2)	H→L+3 (27%)<br>H-5→L+1 (15%)<br>H-2→L+4 (13%)	*ibid*
			226 [0.001] (S_3)	H→L+4 (25%)<br>H-4→L (16%)<br>H-3→L+1 (16%)	$(\pi^*)_{Iso} \leftarrow (\pi)_{Ph+Iso}$
	<240 [n.o.[g]]	208	207 [0.30] (S_6)	H→L (82%)	$(\pi^*)_{Ph+Iso} \leftarrow (\pi)_{Ph+Iso}$
			207 [0.32] (S_7)	H→L+1 (82%)	*ibid*
**4-Me**	475 [0.05]	/	461 [0.00] (S_1)	H→L (54%)<br>H→L+1 (38%)	$(\pi^*)_{TIso} \leftarrow (\pi)_{Ph+TIso}$
**[4-Me]**			461 [0.00] (S_2)	H→L+1 (54%)<br>H→L (38%)	*ibid*
			338 [0.00] (S_1)	H-1→L (31%)<br>H-2→L+1 (30%)<br>H-2→L (15%)<br>H-1→L+1 (14%)	$(\pi^*)_{TIso} \leftarrow (\pi)_{Ph+TIso}$
			338 [0.00] (S_2)	H-1→L+1 (31%)<br>H-2→L (30%)<br>H-1→L (14%)<br>H-2→L+1 (14%)	*ibid*
	380 (sh) [0.2]	/	290 [0.01] (S_8)	H-3→L (61%)<br>H-5→L+1 (14%)<br>H-4→L (12%)	$(\pi^*)_{TIso} \leftarrow (\pi)_{Ph+TIso}$
			289 [0.01] (S_9)	H-3→L+1 (61%)<br>H-5→L (13%)<br>H-4→L+1 (13%)	*ibid*
	300 [32.4]	277	279 [0.40] (S_{10})	H-4→L+1 (35%)<br>H-3→L+1 (29%)<br>H-5→L (20%)	$(\pi^*)_{TIso} \leftarrow (\pi)_{TIso}$
			279 [0.40] (S_{11})	H-4→L (29%)<br>H-3→L (28%)<br>H-5→L+1 (26%)	*ibid*
**6-Me**	440 (sh) [0.02]	/	366 [0.00] (S_1)	H→L (95%)	$(\pi^*)_{TIso} \leftarrow (\pi)_{Ph+TIso}$
**[6-Me]**	380 [0.1]		255 [0.00] (S_2)	H-3→L (90%)	*ibid*
			252 [0.06] (S_3)	H-2→L (35%<br>H-6→L (32%))<br>H-1→L (24%))	*ibid*
	304 (sh) [8.8]	249	252 [0.16] (S_4)	H-1→L (61%)<br>H-2→L (14%)<br>H-6→L (13%)	$(\pi^*)_{C=X} \leftarrow (\pi)_{Ph+TIso}$
	278 [15.1]		247 [0.21] (S_6)	H→L+2 (92%)	$(\pi^*)_{Ph} \leftarrow (\pi)_{Ph+TIso}$

		237(sh)	233[0.19] (S_{12})	H-7→L (71%)<br>H→L (17%)	$(\pi^*)_{TIso}+(\pi^*)_{Ph}\leftarrow(\pi)_{Ph+C=S}$
**7-Me**	440 (sh) [0.06]	/	431 [0.00] (S_1)	H→L (95%)	$(\pi^*)_{TIso} \leftarrow (\pi)_{Ph+TIso}$
**[7-Me]**	380 [0.1]		363 [0.00] (S_2)	H-1→L (64%)<br>H→L+1 (29%)	*ibid*
			308 [0.00] (S_3)	H→L+1 (69%)<br>H-1→L (31%)	*ibid*
			278 [0.001] (S_4)	H-4→L (84%)<br>H-1→L+1 (10%)	*ibid*
	304 [24.7]	277	279 [0.35] (S_5)	H-2→L (93%)	$(\pi^*)_{C=X}\leftarrow (\pi)_{Ph}+(\pi)_{C=S}$
	252 [16.5]	258	258 [0.23] (S_{12})	H→L (26%)	$(\pi^*)_{Ph+TIso} \leftarrow (\pi)_{Ph+TIso}$
**5'** [C1]	470 [0.3]	465	465 [0.00] (S_1)	H→L+1 (44%)<br>H→L (27%)<br>H-3→L+1 (13%)	$(\pi^*)_{TIso} \leftarrow (\pi)_{Ph+TIso}$
**[5]**			465 [0.00] (S_2)	H→L (44%)<br>H→L+1 (27%)<br>H-3→L+1 (13%)	*ibid*
	438 [0.3]	374	374 [0.00] (S_4)	H-1→L (26%)<br>H-2→L (24%)<br>H-2→L+1 (22%)<br>H-1→L+1 (22%)	*ibid*
			373 [0.00] (S_5)	H-2→L (23%)<br>H-1→L+1 (23%)<br>H-2→L+1 (20%)<br>H-1→L (20%)	*ibid*
	351 [167.0]	360	367 [2.36] (S_7)	H→L+2 (39%)<br>H→L+3 (36%)	$(\pi^*)_{Ph+Flu} \leftarrow (\pi)_{Ph+Flu+TIso}$
			367 [2.37] (S_8)	H→L+2 (36%)<br>H→L+3 (39%)	*ibid*
	331 [177.0]	332	331[0.56] (S_{13})	H-2→L+2 (29%)<br>H-1→L+3 (27%)<br>H→L+2 (19%)<br>H-2→L+4 (13%)	$(\pi^*)_{Ph+Flu} \leftarrow (\pi)_{Ph+Flu}$
	/		331 [0.55] (S_{14})	H-2→L+3 (28%)<br>H-1→L+2 (28%)<br>H→L+3 (13%)<br>H-1→L+4 (13%)	*ibid*

[a] excited states calculated after optimisation are all ^1A. [b] Experimental absorption (nm) and extinction coefficients (ε) in 10^3 $M^{-1}.cm^{-1}$. [c] λ_{max} and λ_{cal} are the maximum wavelength obtained from the simulated absorption bands, and the TD-DFT calculated wavelength of a singlet excitation, respectively in nm. [d] Computed oscillator strength. [e] Excited state number. [f] Iso: isocyanurate core, TIso: thioisocyanurate core, Ph: phenyl rings. [g] n.o.: not observed.

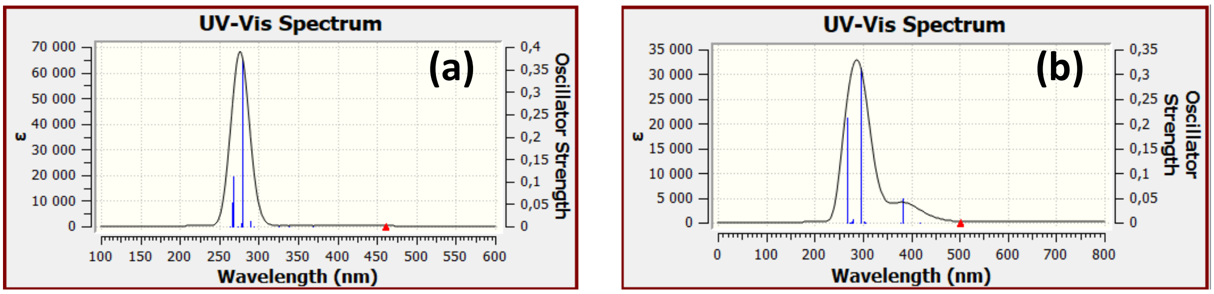

Figure S17. TD-DFT-computed spectra for **4-Me** with dihedral angle of 0° (a) and 60° between the peripheral tolyl groups and the plane of the central heterocycle. In the second case (b), the conformer is destabilized by *ca.* 15 kcal/mol relative to that in (a). A Gaussian broadening function has been applied to the DFT calculated spectra (half-width: 0.185 eV).

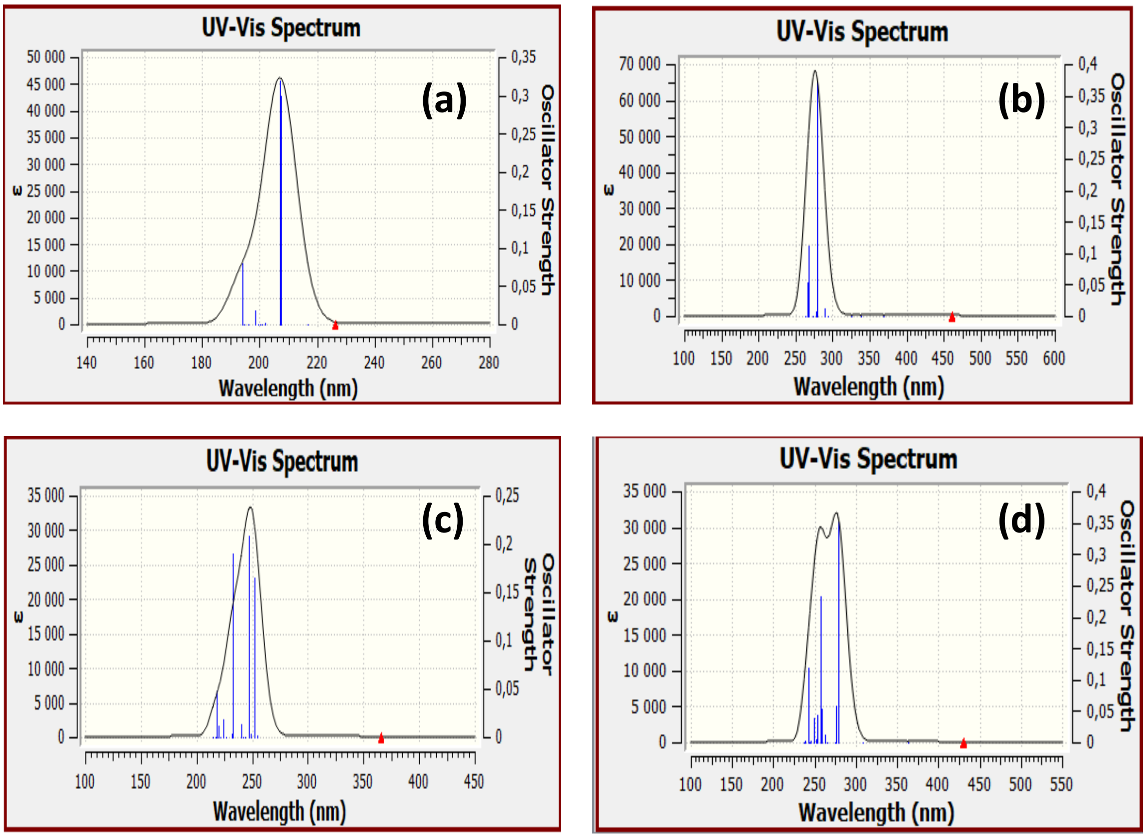

Figure S18. TD-DFT-computed spectra for **1-Me** (a), **4-Me** (b), **6-Me** (c) and **7-Me** (d). A Gaussian broadening function has been applied to the DFT calculated spectra (half-width: 0.185 eV).

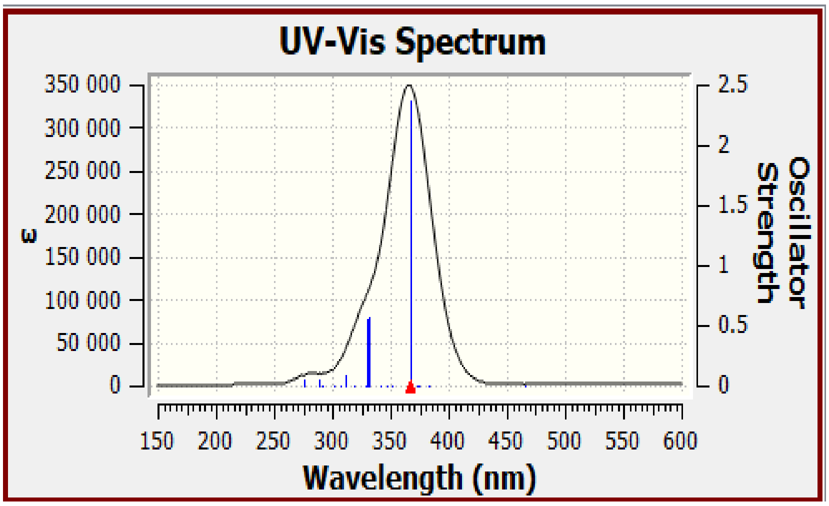

Figure S19. TD-DFT-computed spectra for **5'**. A Gaussian broadening function has been applied to the DFT calculated spectra (half-width: 0.185 eV).

12. Computed energies of the first triplet states for 1-Me, 3', 4-Me, 5', 6-Me and 7-Me

Table S8. Computed energies of the first triplet states (MPW1PW91 / 6-31G*) for **1-Me**, **3'**, **4-Me**, **5'**, **6-Me** and **7-Me** (in eV) relative to S_0.

Cpnd	S_1 [a]	T_1 [b]	$\Delta E(T_1\text{-}S_1)$	$\Delta E(S_1\text{-}S_0)$ Fluorescence	Osillator Strength towards S_0	$\Delta E(S_0\text{-}S_1)$ vertical
**1-Me**	5.31	3.58 [c]	1.73	5.03	0.04	5.48
**3'** [c,d]	3.13	2.31	0.82	3.02	0.00	2.66
**4-Me**	2.55	2.30	0.25	2.42	0.0	2.47
**5'** [d]	3.89	2.41	1.48	3.65	0.00	3.56
**6-Me**	3.00	2.61	0.39	2.06	0.011	3.39
**7-Me**	2.71	2.53	0.18	2.53	0.0	2.88

[a] After vibrational relaxation. [b] Obtained via unresticted calculations (T_1). [c] In **3'** the butyl chains of **3** have been replaced by methyl groups to expedite the DFT calculations. [d] C1 conformation.

13. References

[1] Silverstein, R. M.; Morrill, T. C.; Bassler, G. C., *Spectrometric Identification of Organic Compounds*. John Wiley: New York, 1991.

[2] Bellamy, L. J., *The Infrared Spectra of Complex Molecules*. Methuen: London, 1955.

[3] A. M. Brouwer, *Pure Appl. Chem.* **2011**, *83*, 2213-2228.
